# miR−122−5p Regulates Renal Fibrosis In Vivo

**DOI:** 10.3390/ijms232315423

**Published:** 2022-12-06

**Authors:** Shohei Kaneko, Katsunori Yanai, Hiroki Ishii, Akinori Aomatsu, Keiji Hirai, Susumu Ookawara, Kenichi Ishibashi, Yoshiyuki Morishita

**Affiliations:** 1 Division of Nephrology, First Department of Integrated Medicine, Saitama Medical Center, Jichi Medical University, Saitama 330-8503, Japan; 2 Division of Intensive Care Unit, First Department of Integrated Medicine, Saitama Medical Center, Jichi Medical University, Saitama 330-8503, Japan; 3 Department of Medical Physiology, Meiji Pharmaceutical University, Tokyo 204-8588, Japan

**Keywords:** microRNA, renal fibrosis, end-stage renal disease, chronic kidney disease, miR−122−5p

## Abstract

The role of exogenous microRNAs (miRNAs) in renal fibrosis is poorly understood. Here, the effect of exogenous miRNAs on renal fibrosis was investigated using a renal fibrosis mouse model generated by unilateral ureteral obstruction (UUO). miRNA microarray analysis and quantitative reverse-transcription polymerase chain reaction showed that miR−122−5p was the most downregulated (0.28-fold) miRNA in the kidneys of UUO mice. The injection of an miR−122−5p mimic promoted renal fibrosis and upregulated *COL1A2* and *FN1*, whereas an miR−122−5p inhibitor suppressed renal fibrosis and downregulated *COL1A2* and *FN1*. The expression levels of fibrosis-related mRNAs, which were predicted targets of miR−122−5p, were evaluated. The expression level of *TGFBR2*, a pro-fibrotic mRNA, was upregulated by the miR−122−5p mimic, and the expression level of *FOXO3*, an anti−fibrotic mRNA, was upregulated by the miR−122−5p inhibitor. The protein expressions of TGFBR2 and FOXO3 were confirmed by immunohistochemistry. Additionally, the expression levels of *LC3*, downstream anti-fibrotic mRNAs of *FOXO3*, were upregulated by the miR−122−5p inhibitor. These results suggest that miR−122−5p has critical roles in renal fibrosis.

## 1. Introduction

Chronic kidney disease (CKD) is currently a major public health problem associated with patient mortality [1,2]. The prevalence of CKD worldwide is increasing rapidly [1,2,3]. CKD develops and progresses with hypertension, diabetes, and dyslipidemia [3]. In addition, CKD is associated with an increased risk of cardiovascular disease [4]. Various therapeutic agents have been proposed for the management of patients with CKD, including the management of hypertension, diabetes, and dyslipidemia [3]. However, these therapeutic agents are not specific for the treatment of CKD [3]. Therefore, establishing effective and specific therapeutic agents for CKD is required.

One of the most important pathological features of CKD is renal fibrosis [5,6,7,8,9], which is associated with complex pathological mechanisms including inflammation and apoptosis and is characterized by the accumulation of extracellular matrix in the renal interstitial area [5,6,7,8,9,10]. Renal fibrosis is considered the final and common pathway for the progression of end-stage CKD [5,6,7,8,9,10]. Therefore, establishing a therapeutic strategy for renal fibrosis is considered critical for preventing CKD [5,6,7,8,9]. However, renal fibrosis involves complex processes including many types of cells, such as renal tubular cells, vascular endothelial cells, and bone marrow derived cells. Currently, no effective treatments for renal fibrosis have been established [1,9]. 

Many recent reports have demonstrated the importance of microRNAs (miRNAs), a functional nucleic acid, in disease [11,12]. miRNAs are non-coding small RNAs with a length of 21–25 bases that regulate various pathological conditions and diseases by repressing the 3′ untranslated region of target messenger RNAs (mRNAs) [11,12]. Several studies showed that miRNAs have an important role in tissue fibrosis in various organs [13,14]. In the kidney, several miRNAs have been associated with renal fibrosis, and both anti-fibrotic and pro-fibrotic miRNAs have been identified [3,13,14,15,16,17,18,19,20]. These reports suggest the possibility of exogenic miRNAs as novel therapeutic targets for renal fibrosis. However, few studies have investigated the effects of exogenous miRNAs on renal fibrosis in vivo [9,21,22,23]. Therefore, the investigation of miRNAs that may regulate renal fibrosis is critical for identifying potential therapeutic agents for renal fibrosis.

In this study, we screened miRNAs in a renal fibrotic mouse model to identify those associated with renal fibrosis in vivo. We also investigated the effects and mechanisms of the candidate miRNAs in the renal fibrotic mouse model.

## 2. Results

### 2.1. Profiling of miRNAs in Renal Fibrosis

**Figure 1:**
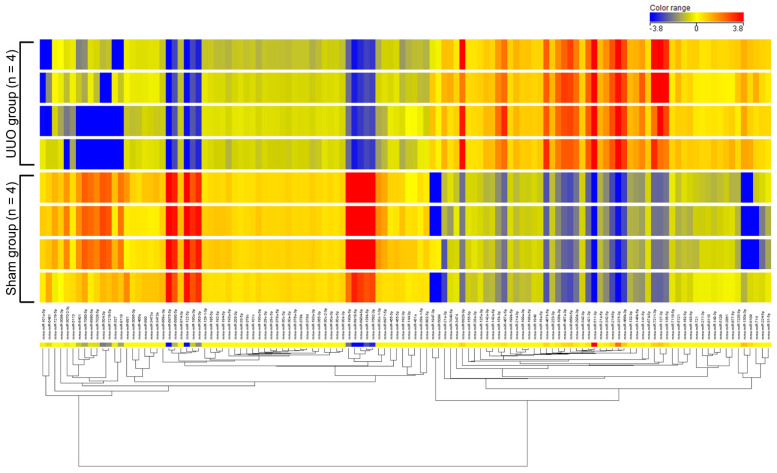
Heat map of differentially expressed miRNAs in fibrotic kidneys. Heat map shows hierarchical clustering and systemic variations in the expression levels of miRNAs in the UUO group (n = 4) and sham group (n = 4). Red, increased expression; blue, decreased expression. Abbreviations: UUO, unilateral ureteral obstruction; miR, microRNA.

**Figure 2:**
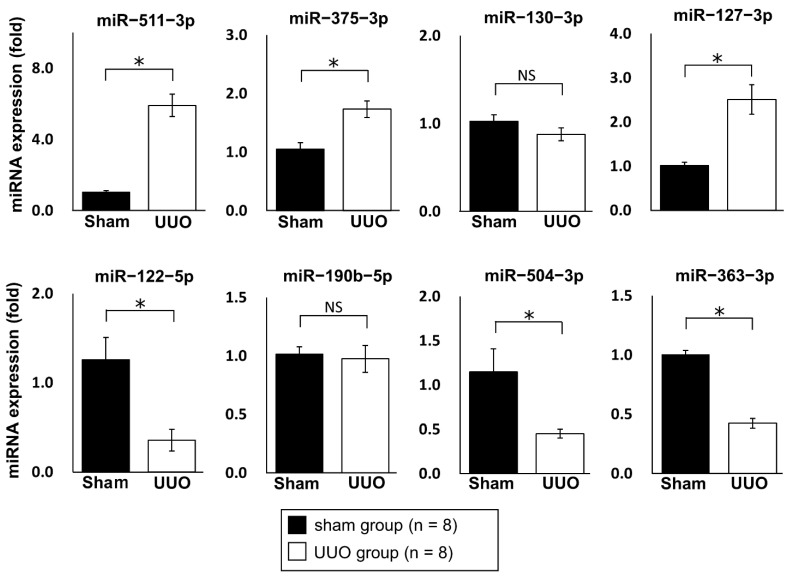
Regulated miRNAs in fibrotic kidneys. The expression levels of eight miRNAs (miR−511−3p, −375−3p, −130−3p, −127−3p, −122−5p, −190b−5p, −504−3p, and −363−3p) in the UUO group (n = 8) and sham group (n = 8) were determined by qRT-PCR. Relative expression levels are shown as mean values and standard errors. Abbreviations: miR, microRNA; UUO, unilateral ureteral obstruction; qRT-PCR, quantitative real-time reverse-transcription polymerase chain reaction; NS, not significant. * *p* < 0.05.

We examined differentially expressed miRNAs in renal fibrosis by analyzing the expression of 1915 miRNAs [miRBase database release 21.0; www.mirbase.org (accessed on 03 February 2016)] in a renal fibrotic mouse model established by unilateral ureteral obstruction (UUO) using miRNA microarray analysis. Sixteen mice were randomly divided into two groups: UUO group (n = 8) and sham group (n = 8). After surgery, kidneys were collected, miRNAs were extracted, and eight samples [UUO group (n = 4) and sham group (n = 4)] with especially high quality were selected (details are given in the Materials and Methods). The expression levels of miRNAs were compared between the two groups. The results showed that 109 miRNAs were upregulated more than 2-fold in the kidneys of UUO mice compared with sham surgery mice, and 113 miRNAs were downregulated less than 0.5-fold in the kidneys of UUO mice compared with sham surgery mice (Table 1, Figure 1). The top 10 upregulated miRNAs and bottom 10 downregulated miRNAs were selected as candidates for further analysis. Of these 20 miRNAs, those previously reported to be associated with renal fibrosis were excluded. The remaining eight miRNAs (miR−511−3p, −375−3p, −130−3p, −127−3p, −122−5p, −190b−5p, −504−3p, and −363−3p) were quantitatively evaluated by quantitative real-time reverse−transcription polymerase chain reaction (qRT-PCR) (Figure 2). In this process, sixteen mice [UUO group (n = 8) or sham group (n = 8)] described above were used as samples. qRT-PCR analysis showed that three miRNAs (miR−511−3p, −375−3p, and −127−3p) were significantly upregulated and three miRNAs (miR−122−5p, −504−3p, and −363−3p) were significantly downregulated in the kidneys of UUO mice compared with control mice. The expression level of miR−122−5p was the most highly downregulated miRNA (0.28-fold downregulated in UUO mice compared with control mice). Therefore, we focused on miR−122−5p in subsequent experiments.

### 2.2. Regulation of Renal Fibrosis by a Mimic and Inhibitor of miR−122−5p 

**Figure 3:**
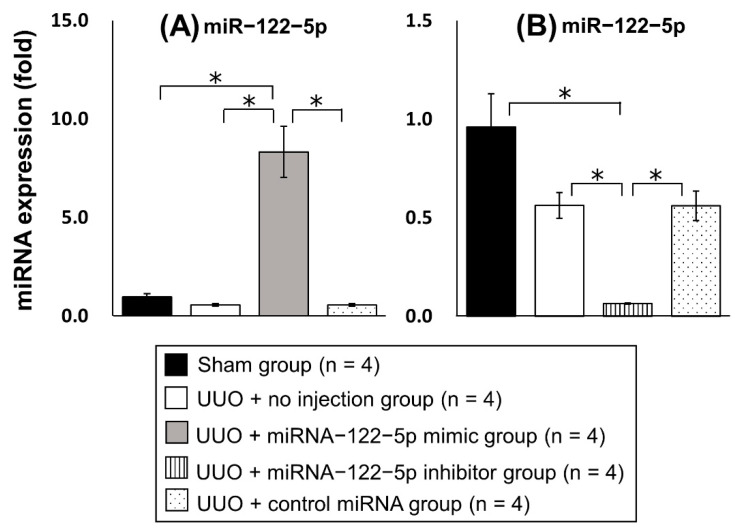
Modulation of miR−122−5p expression by miR−122−5p-mimic and -inhibitor. The expression levels of miR−122−5p were determined by qRT−PCR. Relative expression levels are shown as mean values and standard errors. (**A**) Relative expression levels were compared between the four groups: sham group (n = 4), UUO + no injection group (n = 4), UUO + miR−122−5p mimic group (n = 4), and UUO + control miRNA group (n = 4). (**B**) Relative expression levels were compared between the four groups: sham group (n = 4), UUO + no injection group (n = 4), UUO + miR−122−5p inhibitor group (n = 4), and UUO + control miRNA group (n = 4). Abbreviations: miR, microRNA; UUO, unilateral ureteral obstruction; qRT-PCR, quantitative real-time reverse-transcription polymerase chain reaction. * *p* < 0.05.

**Figure 4:**
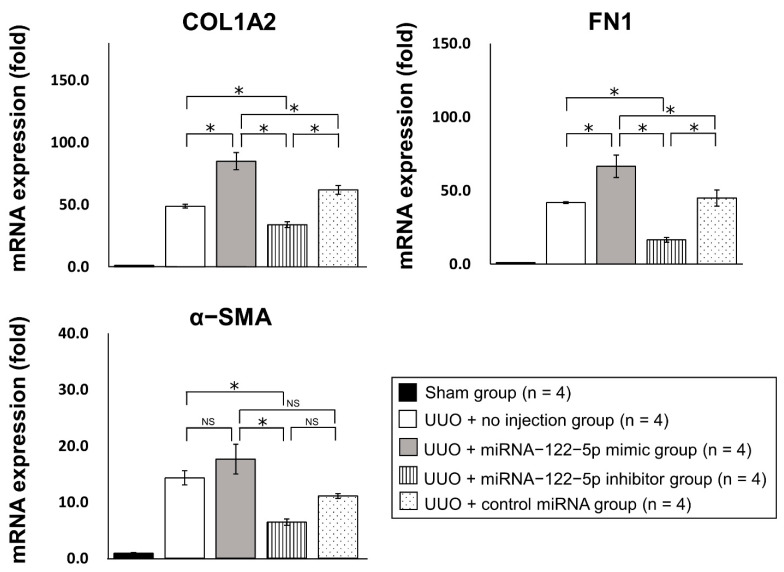
The effects of an miR−122−5p mimic and inhibitor on the severity of renal fibrosis. The expression levels of three mRNAs (*COL1A2*, *FN1*, and *α-SMA*) were determined by qRT-PCR. Relative expression levels are shown as mean values and standard errors. Relative expression levels of mRNAs were compared between the four groups: UUO + no injection group (n = 4), UUO + miR−122−5p mimic group (n = 4), UUO + miR−122−5p inhibitor group (n = 4), and UUO + control miRNA group (n = 4). Abbreviations: miR, microRNA; UUO, unilateral ureteral obstruction; mRNA, messenger RNA; COL1A2, collagen 1A2; FN1, fibronectin 1; α-SMA, α-smooth muscle actin; qRT-PCR, quantitative real-time reverse-transcription polymerase chain reaction. NS, not significant. * *p* < 0.05.

**Figure 5:**
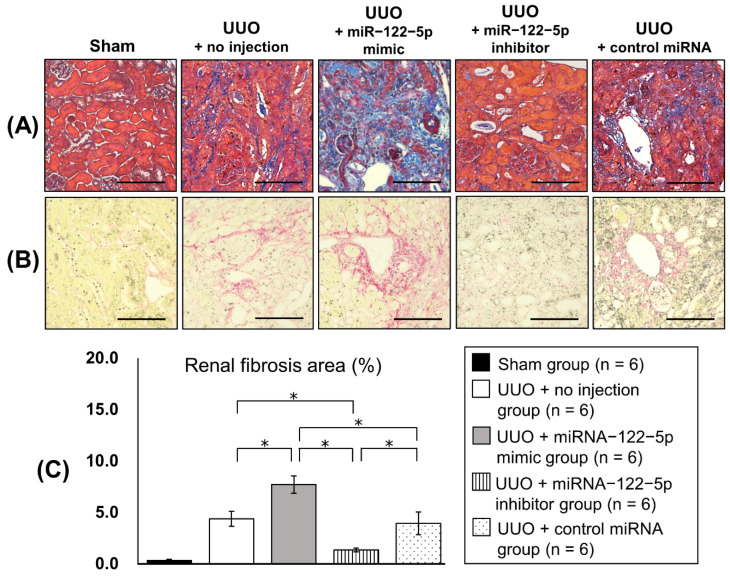
Kidney histology. Severity of renal fibrosis was evaluated by AZAN staining and Sirius red staining. (**A**) Representative images of AZAN staining of kidney sections. (**B**) Representative images of Sirius red staining of kidney sections. (**C**) Renal fibrosis area was evaluated by Sirius red staining. Fibrotic areas were compared between the four groups: UUO + no injection group (n = 6), UUO + miR−122−5p mimic group (n = 6), UUO + miR−122−5p inhibitor group (n = 6), and UUO + control miRNA group (n = 6). Scale bar = 100 µm. * *p* < 0.05. Abbreviations: miR, microRNA; UUO, unilateral ureteral obstruction.

**Figure 6:**
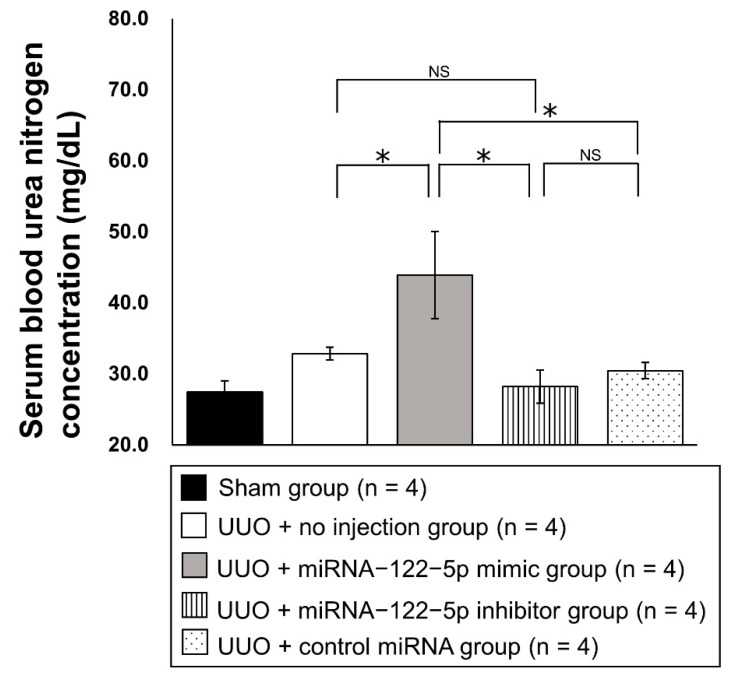
Serum blood urea nitrogen concentrations. Serum blood urea nitrogen concentrations (mg/dL) were compared between the four groups: UUO + no injection group (n = 4), UUO + miR−122−5p mimic group (n = 4), UUO + miR−122−5p inhibitor group (n = 4), and UUO + control miRNA group (n = 4). Abbreviations: miR, microRNA; UUO, unilateral ureteral obstruction; NS, not significant. * *p* < 0.05.

Twenty new mice were randomly divided into five groups: sham group (n = 4), UUO + no injection group (n = 4), UUO + miR−122−5p mimic group (n = 4), UUO + miR−122−5p inhibitor group (n = 4), and UUO + control miRNA group (n = 4) (details are given in the Materials and Methods). After each treatment and surgery, kidneys and serum were collected. The modulation of miR−122−5p expression levels by an miR−122−5 mimic and miR−122−5 inhibitor was confirmed by qRT−PCR; the miR−122−5p mimic significantly enhanced the expression of miR−122−5p, and the miR−122−5p inhibitor significantly suppressed the expression of miR−122−5p (Figure 3). Although the expression of miR−122−5p in the UUO + no injection group showed a decreasing trend compared with the sham group, this did not reach statistical significance (Supplementary Figure S1). We also confirmed that contralateral kidneys showed no change in response to the overexpression or inhibition of miR−122−5p. Next, we evaluated the expression of three genes that are increased in renal fibrosis [collagen 1A2 (*COL1A2*), fibronectin 1 (*FN1*), and α-smooth muscle actin (*α-SMA*)]. The expressions of *COL1A2*, *FN1*, and *α-SMA* were significantly enhanced in the UUO + no injection group compared with the sham group (Supplementary Figure S2). *COL1A2* and *FN1* were upregulated in the UUO + miR−122−5p mimic group compared with the UUO + control miRNA group and UUO + no injection group. In contrast, *COL1A2* and *FN1* mRNAs were downregulated in the UUO + miR−122−5p inhibitor group compared with the UUO + control miRNA group and UUO + no injection group (Figure 4). Although a similar tendency was observed with the expression of *α-SMA*, there was no statistical significance. Renal fibrosis was enhanced in the UUO + miR−122−5p mimic group, as shown by AZAN staining and Sirius red staining, whereas renal fibrosis was suppressed in the UUO + miR−122−5p inhibitor group (Figure 5). The renal fibrosis area in the UUO + no injection group was significantly larger than that in the sham group (Supplementary Figure S3). The renal fibrosis area in the UUO + miR−122−5p mimic group was larger than that in the UUO + control miRNA group and UUO + no injection group, whereas the area was reduced in the UUO + miR−122−5p inhibitor group compared with the UUO + control miRNA group and UUO + no injection group (Figure 5). Serum blood urea nitrogen (BUN) concentrations were significantly increased in the UUO + no injection group compared with the sham group (Supplementary Figure S4). Serum BUN concentrations were significantly increased in the UUO + miR−122−5p mimic group compared with the UUO + control miRNA group and UUO + no injection group. Although a lower trend of serum BUN concentration in the UUO + miR−122−5p inhibitor group was observed, the difference was not significant compared with the UUO + control miRNA group and UUO + no injection group (Figure 6). 

### 2.3. Mechanism of the Modulation of Renal Fibrosis by miR−122−5p

**Figure 7:**
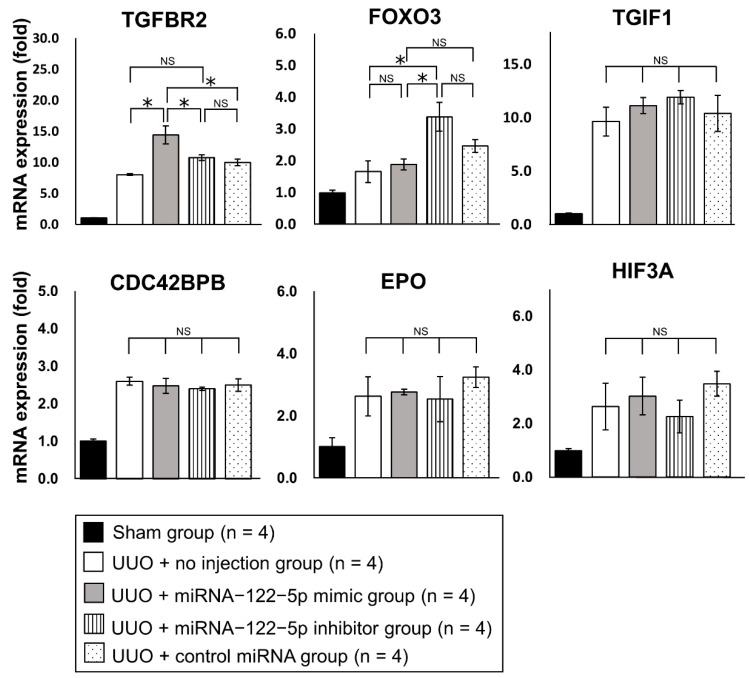
The effects of miR−122−5p on fibrosis-related mRNAs. The expression levels of six mRNAs (*TGFBR2*, *FOXO3*, *TGIF1*, *CDC42BPB*, *EPO*, and *HIF3A*) were determined by qRT-PCR. Relative expression levels are shown as mean values and standard errors. Relative expression levels of mRNAs were compared between the four groups: UUO + no injection group (n = 4), UUO + miR−122−5p mimic group (n = 4), UUO + miR−122−5p inhibitor group (n = 4), and UUO + control miRNA group (n = 4). Abbreviations: miR, microRNA; mRNA, messenger RNA; UUO, unilateral ureteral obstruction; qRT-PCR, quantitative real-time reverse-transcription polymerase chain reaction; TGFBR2, transforming growth factor, beta receptor II; FOXO3, forkhead box O3; TGIF1, Homeobox protein TGIF1; CDC42BPB, CDC42 binding protein kinase beta; EPO, erythropoietin; HIF3A, hypoxia-inducible factor 3 alpha; NS, not significant. * *p* < 0.05.

**Figure 8:**
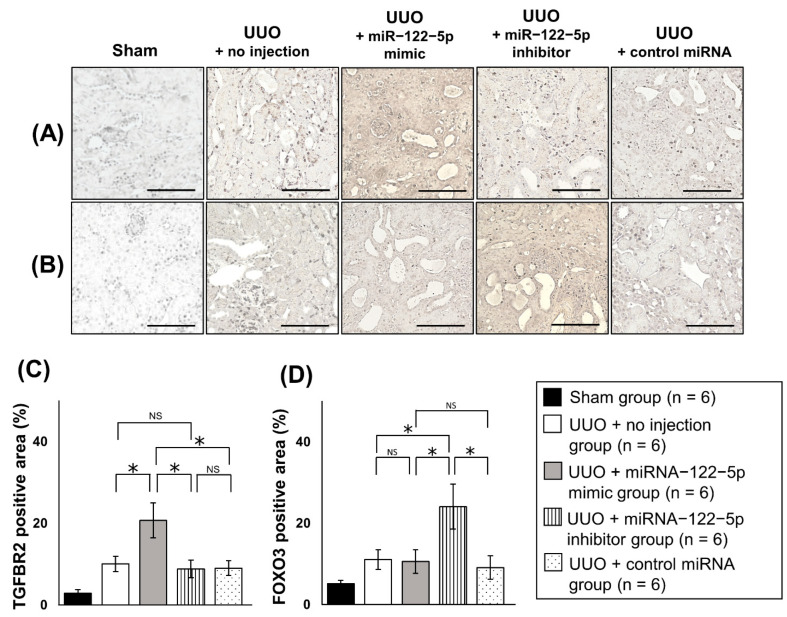
IHC analysis. The protein levels of TGFBR2 and FOXO3 were evaluated. Representative images of IHC to detect (**A**) TGFBR2 and (**B**) FOXO3 in kidney sections. Comparison of (**C**) TGFBR2 and (**D**) FOXO3 positive areas (brown stain). IHC positive areas were quantitatively evaluated and compared between the four groups: UUO + no injection group (n = 6), UUO + miR−122−5p mimic group (n = 6), UUO + miR−122−5p inhibitor group (n = 6), and UUO + control miRNA group (n = 6). Abbreviations: IHC, immunohistochemistry; miR, microRNA; UUO, unilateral ureteral obstruction; TGFBR2, transforming growth factor, beta receptor II; FOXO3, forkhead box O3; NS, not significant. Scale bar = 100 µm. * *p* < 0.05.

**Figure 9:**
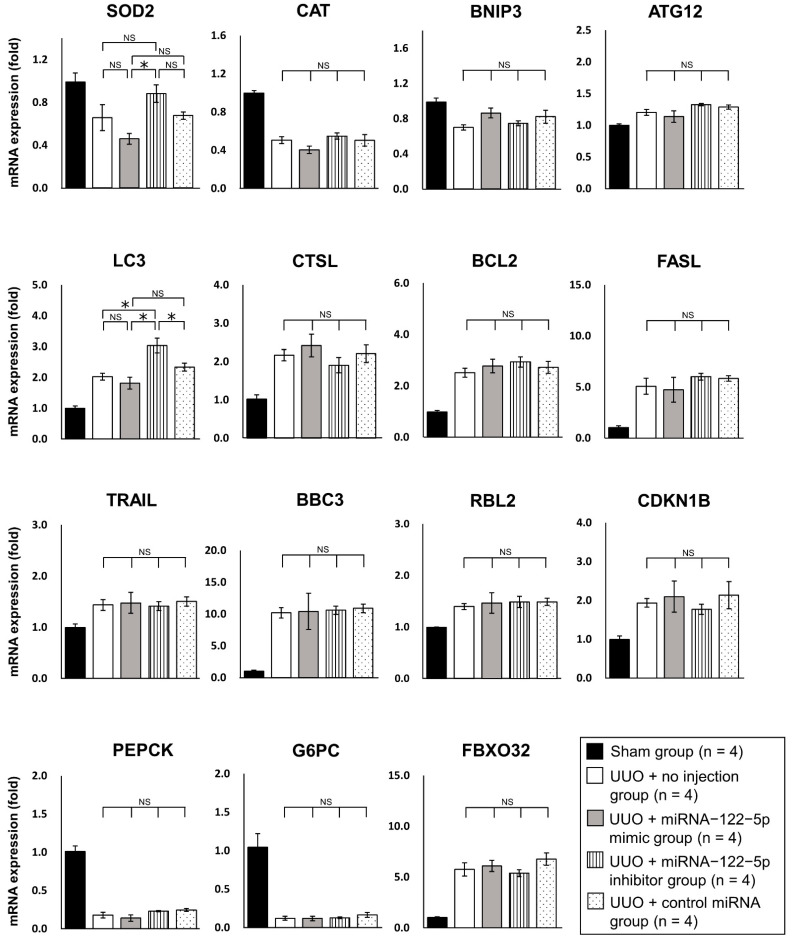
Analysis of downstream factors of FOXO3. The expression levels of 15 genes downstream of FOXO3 *(SOD2, CAT, BNIP3, ATG12, LC3, CTSL, BCL2, FASL, TRAIL, BBC3, RBL2, CDKN1B, PEPCK, G6PC,* and *FBXO32*) were evaluated by qRT-PCR. Relative expression levels of genes were compared between the four groups: UUO + no injection group (n = 4), UUO + miR−122−5p mimic group (n = 4), UUO + miR−122−5p inhibitor group (n = 4), and UUO + control miRNA group (n = 4). Relative expression levels are shown as mean values and standard errors. Abbreviations: miR, microRNA; UUO, unilateral ureteral obstruction; qRT-PCR, quantitative real-time reverse-transcription polymerase chain reaction; SOD2, superoxide dismutase 2, mitochondrial; CAT, catalase; BNIP, BCL2/adenovirus E1B 19 kDa protein-interacting protein 3; ATG12, autophagy related 12; LC3, microtubule-associated proteins 1A/1B light chain 3B; CTSL, cathepsin L1; BCL2, B-cell lymphoma 2; FASL, fas ligand; TRAIL, TNF-related apoptosis-inducing ligand; BBC3, Bcl-2-binding component 3; RBL2, retinoblastoma-like protein 2; CDKN1B, cyclin-dependent kinase inhibitor 1B; PEPCK, phosphoenolpyruvate carboxykinase; G6PC, glucose-6-phosphatase, catalytic subunit; FBXO32, F-box only protein 32; NS, not significant. * *p* < 0.05.

To analyze the mechanism by which miR−122−5p modulated renal fibrosis, we investigated potential target mRNAs for miR−122−5p. Five mRNAs including forkhead box O3 (*FOXO3*), homeobox protein TGIF1 (*TGIF1*), CDC42 binding protein kinase beta (*CDC42BPB*), erythropoietin (*EPO*), and hypoxia−inducible factor 3 alpha (*HIF3A*) mRNAs were predicted to be target mRNAs of miR−122−5p by computer analysis using TargetScan [http://www.targetscan.org/vert_72/ (accessed on 21 May 2020)]. Transforming growth factor beta receptor II (*TGFBR2*) was added as another candidate because *TGFBR2* was previously reported to be modulated by miR−122−5p and was associated with skeletal muscle fibrosis [24]. We examined the expression levels of these six genes in kidney samples from each of the five treatment groups (Figure 7). The expression levels of *TGFBR2*, *TGIF1*, and *CDC42BPB* were significantly upregulated in the UUO + no injection group compared with the sham group. Although the expression levels of *FOXO3*, *EPO*, and *HIF3A* showed an increasing trend in the UUO + no injection group, there was no significant difference compared with the sham group (Supplementary Figure S5). The expression level of *TGFBR2* (pro-fibrotic mRNA) was significantly upregulated in the UUO + miR−122−5p mimic group compared with the UUO + control miRNA group and UUO + no injection group. The expression level of *FOXO3* (anti-fibrotic mRNA) was significantly elevated in the UUO + miR−122−5p inhibitor group compared with the UUO + no injection group. In comparison with the UUO + control miRNA group, no significant difference was observed although there was an increasing trend of *FOXO3* levels in the UUO + miR−122−5p inhibitor group (Figure 7). Because it was difficult to reach conclusions from the mRNA (*TGFBR2* and *FOXO3*) analysis alone, the protein expressions of TGFBR2 and FOXO3 were evaluated by immunohistochemistry (IHC). We found that FOXO3 and TGFBR2 were ubiquitously expressed in the kidney when assessed by IHC (Figure 8). The TGFBR2 positive and FOXO3 positive areas in the UUO + no injection group were significantly larger than that in the sham group (Supplementary Figure S6). The TGFBR2 positive area in the UUO + miR−122−5p mimic group was larger than that in the UUO + control miRNA group and UUO + no injection group. Additionally, the FOXO3 positive area in the UUO + miR−122−5p inhibitor group was larger than that in the UUO + control miRNA group and UUO + no injection group (Figure 8). In previous studies, FOXO3 was reported to have a protective role against renal fibrosis and CKD [25,26,27,28]. Although the mechanism involved is not entirely clear, it has been suggested that FOXO3 alleviates cellular damage in the tubules through antioxidant activity and autophagy activation [25,26,27,28]. For this reason, we focused on FOXO3 in this study. The KEGG PATHWAY Database [https://www.genome.jp/kegg/pathway.html (accessed on 3 August 2021)] was used to predict downstream antifibrotic mRNA of FOXO3 to investigate the mechanism involved in its anti-fibrotic effects. Expression levels of 15 predicted mRNAs [superoxide dismutase 2, mitochondrial (*SOD2*), catalase (*CAT*), BCL2/adenovirus E1B 19 kDa protein-interacting protein 3 (*BNIP3*), autophagy related 12 (*ATG12*), microtubule-associated proteins 1A/1B light chain 3B (*LC3*), cathepsin L1 (*CTSL*), B-cell lymphoma 2 (*BCL2*), Fas ligand (*FASL*), TNF-related apoptosis-inducing ligand (*TRAIL*), Bcl-2-binding component 3 (*BBC3*), retinoblastoma-like protein 2 (*RBL2*), cyclin-dependent kinase inhibitor 1B (*CDKN1B*), phosphoenolpyruvate carboxykinase (*PEPCK*), glucose-6-phosphatase, catalytic subunit (*G6PC*), and F-box only protein 32 (*FBXO32*)] were evaluated using qRT-PCR. In the UUO + no injection group, the expression levels of *ATG12*, *LC3*, *CTSL*, *BCL2*, *FASL*, *TRAIL*, *BBC3*, *RBL2*, *CDKN1B*, and *FBXO32* were significantly upregulated compared with the sham group. In contrast, the expression levels of *CAT*, *BNIP3*, *PEPCK*, and *G6PC* were significantly downregulated in the UUO + no injection group compared with the sham group (Supplementary Figure S7). The expression level of *LC3* (autophagy related mRNA) was significantly upregulated in the miR−122−5p + inhibitor group compared with the UUO + control miRNA group and UUO + no injection group. Although the expression level of *SOD2* (antioxidant related mRNA) in the UUO + miR−122−5p inhibitor group was not significantly upregulated compared with the UUO + control miRNA group and UUO + no injection group, there was a significant difference in levels between the UUO + miR−122−5p inhibitor group and UUO + miR−122−5p mimic group (Figure 9).

## 3. Discussion

Our study showed that miR−122−5p modulates renal fibrosis. The main study findings were: (i) the expression of miR−122−5p was most downregulated in fibrotic kidneys compared with control kidneys as assessed by microarray analysis; (ii) the injection of miR−122−5p promoted renal fibrosis and upregulated TGFBR2 (pro-fibrotic factor); and (iii) miR−122−5p suppressed renal fibrosis and upregulated FOXO3 and *LC3* (anti-fibrotic factors). To the best of our knowledge, this is the first report to show that miR−122−5p modulates renal fibrosis in vivo. Although certain miRNAs were reported to be associated with renal fibrosis [3,13,14,15,16,17,18,19,20], there have been few reports on the effects of exogenous miRNA modulation on renal fibrosis. Therefore, our findings regarding the exogenous modulation of miR−122−5p expression and its effect on renal fibrosis are considered significant.

Previously, several miRNAs were reported to be associated with renal fibrosis [3,13,14,15,16,17,18]—the expression levels of miR−21, −22, −135a, −150, −155, −184, −214, −215, −216a, −324, −433, and −1207 were upregulated in fibrotic kidneys [18,29,30,31,32]. In contrast, the expression levels of let-7, miR−29, −30, −34, −152, −181, −194, −200, and −455 were downregulated in fibrotic kidneys [18,21,33,34,35]. In this study, we confirmed that the expression levels of three miRNAs (miR−511−3p, −375−3p, and −127−3p) were significantly upregulated, and the expression levels of three miRNAs (miR−122−5p, −504−3p, and −363−3p) were significantly downregulated, in fibrotic kidneys by qRT−PCR. None of these six miRNAs had been reported to be associated with renal fibrosis at the time we conducted this study. The reasons for our discovery of these novel miRNAs associated with renal fibrosis might include differences in experimental design (in vivo or in vitro experiments), the termination period of UUO mice, and the datasets used for microarray analyses. We selected miR−122−5p for in-depth analyses because it was the most downregulated miRNA in fibrotic kidneys. Several previous studies of miR−122−5p reported its involvement in tissue fibrosis, but not in the kidney. Sun et al. reported the downregulation of miR−122−5p in fibrotic skeletal muscle tissue and showed that the overexpression of miR−122−5p suppressed skeletal muscle fibrosis [24]. Similarly, Halász et al. reported a negative correlation between miR−122−5p expression levels and the severity of liver fibrosis [36]. The other five miRNAs (miR−511−3p, −375−3p, −127−3p, 504−3p, and −363−3p) we identified might also be interesting targets for future research. In particular, miR−375−3p was reported to be associated with liver fibrosis and cardiac fibrosis [37,38]. In addition, miR−127−3p might be involved in tissue repair in ischemic kidney models [39]. 

In this study, a mimic or inhibitor of miR−122−5p was combined with polyethyleneimine nanoparticles (PEI-NPs) to enable the reagent to traffic to the kidneys (details are presented in Section 4.6.). The efficacy and safety of PEI−NPs were previously described [9]. Our histological and quantitative analysis confirmed that the miR−122−5p mimic or inhibitor with PEI-NPs modulated renal fibrosis. The serum BUN concentration was significantly increased by miR−122−5p mimic injection compared with the other groups. In contrast, a lower trend of serum BUN concentration in the UUO + miR−122−5p inhibitor group was observed; however, the difference was not significant when compared with the UUO + control miRNA group and UUO + no injection group. These results may be explained by the decreasing range of BUN induced by the miR−122−5p inhibitor was smaller than the increasing range of BUN induced by the miR−122−5p mimic. Increasing the sample size might help reach statistically significant differences, and further investigations will be interesting.

The study findings suggested that TGFBR2 (a pro-fibrotic factor) might be involved in the mechanism by which the miR−122−5p mimic promotes renal fibrosis and FOXO3 (an anti-fibrotic factor) might be involved in the mechanism by which the miR−122−5p inhibitor suppresses renal fibrosis. The TGF-β/Smad signaling pathway, of which TGFBR2 is an important constituent, has a central role in tissue fibrosis [40]. TGFBR2 was reported to be downregulated by the overexpression of miR−122−5p in fibrotic skeletal muscle tissue, leading to the suppression of fibrotic changes [24]. In contrast with our expectations, we found that the miR−122−5p mimic enhanced renal fibrosis with the upregulation of TGFBR2. These results suggest that miR−122−5p might have different roles in different organs. Although miRNAs generally function to silence gene expression [11,12], our results show that the miRNA−122−5p mimic upregulated *TGFBR2* expression. This suggests there is an as yet unidentified and intervening pathway between miR−122−5p and *TGFBR2*. FOXO3 was reported to have anti−fibrotic functions in various organs including the kidney (described later) [27,41]. We found that the upregulation of FOXO3 by the miRNA−122−5p inhibitor had therapeutic potential for renal fibrosis. Despite *FOXO3* being identified as a potential target of miR−122−5p by computer analysis (TargetScan), *FOXO3* was not decreased in the UUO + miR−122−5p mimic group compared with the UUO + control miRNA group and UUO + no injection group. This suggests there may be an unknown pathway between miR−122−5p and *FOXO3*. IHC showed that FOXO3 was ubiquitously expressed in the kidneys, which is consistent with previous studies reporting FOXO3 is expressed ubiquitously (nuclear and cytoplasmic regions) [25]. In this study, IHC also showed that TGFBR2 was ubiquitously expressed in the kidneys. However, the localization of TGFBR2 could not be determined. TGFBR are thought to be present on the cell membrane; however, it was previously reported to be transported intracellularly [42]. Therefore, further studies regarding the localization of TGFBR2 in kidneys are needed. 

As mentioned in the Results section, the beneficial effects of FOXO3 on renal fibrosis and CKD were recently reported [25,26,27,28]. FOXO3 appears to be protective against renal fibrosis and CKD [25,26,27,28]. However, the details remain unclear. For this reason, we focused on FOXO3 for further exploration in this study. Cell stress, insulin signals, growth factors, and hypoxia are upstream physiological signals that regulate the expression level of FOXO3 [27,43,44]. As mentioned earlier, FOXO3 has anti-fibrotic effects in various organs [27,41], which might be mediated by antioxidant responses, autophagy, and the inhibition of myofibroblast proliferation [26,27,28]. However, there have been few reports describing the antifibrotic mechanisms of FOXO3 in the kidney compared with the liver and lung [41]. Therefore, a deeper understanding and confirmation of how FOXO3 ameliorates renal fibrosis are needed. In our study, KEGG PATHWAY Database analysis showed that several genes (*SOD2*, *CAT*, *BNIP3*, *ATG12*, *LC3*, *CTSL*, *BCL2*, *FASL*, *TRAIL*, *BBC3*, *RBL2*, *CDKN1B*, *PEPCK*, *G6PC*, and *FBXO32*) were downstream of FOXO3. The expression of *LC3* was significantly upregulated in the UUO + miR−122−5p inhibitor group compared with the UUO + control miRNA group and UUO + no injection group. Because *LC3* is involved in autophagy responses, our results are consistent with previous reports explaining the mechanisms whereby FOXO3 ameliorated renal fibrosis and CKD [25,26,27,28]. LC3 is a key regulator of autophagy [45]. Previously, autophagy was reported to be induced in fibrotic kidneys and to have a protective effect against renal fibrosis [46,47]. Moreover, autophagy-deficiency (LC3-deficiency) promoted renal fibrosis [45]. Although its involvement in podocyte self-repair has been suggested as a mechanism by which autophagy ameliorates renal fibrosis [45,48], the details remain unclear. Enhanced autophagy against renal fibrosis is a potential future therapeutic target and an interesting area for future research [49]. In this study, the expression level of *SOD2* in the UUO + miR−122−5p inhibitor group was not significantly altered compared with the UUO + control miRNA group and UUO + no injection group. However, there was a significant difference in *SOD2* expression between the UUO + miR−122−5p inhibitor group and UUO + miR−122−5p mimic group (Figure 9). Although we could not reach conclusions from these results, they suggest that miR−122−5p mimic and inhibitor may involve the expression levels of *SOD2*. *SOD2* is a widely known antioxidant gene that reduces oxidative stress and has protective functions against renal fibrosis [41]. Previously, Yoon et al. reported that tempol (a chemically synthesized antioxidant) increased *SOD2* and ameliorated renal fibrosis in UUO mice via the modulation of FOXO3 signaling [26]. Additionally, Yang et al. reported that fucoxanthin (a marine carotenoid) enhanced FOXO3 expression and increased SOD2 in cultured mesangial cells [50]. These findings are consistent with our results. The relationship between FOXO3 and *SOD2* was not proven in this study. Further study is needed to clarify the relationship between FOXO3 and *SOD2* in the future. There have been several reports on the effects of LC3 and SOD2 on CKD and its causative diseases, including human studies (Table 2). Based on the results of our study alone, it is difficult to conclude that FOXO3 has a beneficial effect on renal fibrosis and CKD. However, our results are consistent with previous reports and support the reported positive effect of FOXO3 on renal fibrosis and CKD [25].

There were several limitations in this study. First, this study used a small sample size. Second, we did not clarify the detailed mechanism regarding the upregulation of *TGFBR2* by the miR−122−5p mimic. Third, we did not investigate its effects on organs other than the kidney. Fourth, this was an animal study and thus the effects of the miR−122−5p mimic on humans are unknown. The therapeutic effect of miR−122−5p on renal fibrosis should be explored in future large-scale studies.

In conclusion, this study showed that miR−122−5p was downregulated in the fibrotic kidneys of a mouse renal fibrosis model. An miR−122−5p mimic enhanced renal fibrosis with the increased expression of TGFR2, whereas an miR−122−5p inhibitor suppressed renal fibrosis with the increased expression of FOXO3 (with the upregulation of the downstream genes, *LC3*). These results indicate that miR−122−5p may have a critical role in renal fibrosis.

## 4. Materials and Methods

### 4.1. Ethical Approval 

All protocols for animal experiments were approved by the Animal Ethics Committee of Jichi Medical University (17012-03) and complied with the guidelines of Use and Care of Experimental Animals of Jichi Medical University.

### 4.2. Renal Fibrotic Mouse Model

Male C57BL/6 mice (aged 8 weeks and weighing 20–25 g) were purchased from Tokyo Laboratory Animals Science Co. (Tokyo, Japan). Mice were maintained under antiviral and antibody-free micro-isolator conditions at 19–21 °C, with 12-h light-dark cycles. UUO surgery was performed to establish the mouse renal fibrotic model as previously described [64,65,66]. In brief, the left ureter was surgically double ligated with 4-0 silk at the lower pole level of the kidney under inhalation anesthesia with isoflurane. The mice were euthanized 7 or 10 days after UUO surgery, depending on the experiment. The left kidney that developed hydronephrosis was collected for pathological and molecular analyses. Blood samples were drawn from the inferior vena cava. In the control group, mice were treated with sham surgery. Sham surgery was the same procedure as UUO except for the ureter ligation.

### 4.3. miRNA Microarray Analysis, Data Processing, and Statistical Analysis

Sixteen C57BL/6 mice were purchased for miRNA microarray analysis and randomly divided into two groups: UUO group (n = 8) or sham group (n = 8). Seven days after UUO surgery or sham surgery, kidney tissue was obtained. Total RNA was extracted using the miRNeasy Mini Kit (Qiagen, Venlo, The Netherlands). Microarray analysis was conducted by Hokkaido System Science (Hokkaido, Japan). A quality check of the RNA was performed using an Agilent 2100 BioAnalyzer series II (Agilent Technologies, Santa Clara, CA, USA) and eight samples of particularly high quality were selected [UUO group (n = 4) and sham group (n = 4)]. The detailed procedure of miRNA microarray analysis was previously described [67]. In brief, the dephosphorylation and hybridization of total RNA were performed using the miRNA Complete Labeling Reagent and Hyb kit (Agilent Technologies) according to the manufacturer’s instructions. Glass slides were washed with Gene Expression Wash Buffer (Agilent Technologies). Data scanning was performed using an Agilent Technologies Microarray Scanner (Agilent Technologies). Obtained data were quantified with Agilent Feature Extraction software 10.7.3.1 (Agilent Technologies) and standardized with GeneSpring 12.1 (Agilent Technologies). For statistical analysis, the Student’s *t* test was used to compare the two groups (UUO group and sham group). *p* < 0.05 was considered to represent statistical significance.

### 4.4. qRT-PCR

qRT-PCR was conducted for the quantitative evaluation of the expression of miRNA and mRNA as previously described [64]. In brief, for the analysis of miRNAs, total RNA was extracted from homogenized kidney samples using the miRNeasy Mini Kit (Qiagen), and 1 µg of total RNA was reverse transcribed to complementary DNA (cDNA) using the miScript II RT Kit (Qiagen). qRT-PCR was performed using a miScript SYBR Green PCR Kit (Qiagen) with miRNA-specific primers (Qiagen) for miR−511−3p, −375−3p, −130−3p, −127−3p, −122−5p, −190b−5p, −504−3p, and −363−3p (Qiagen). For the analyses of mRNA, mRNA was extracted from homogenized kidney samples using the RNeasy Mini Kit (Qiagen) and 1 µg of total RNA was reverse transcribed to cDNA using the SuperScript III First-Strand Synthesis SuperMix (Thermo Fisher Scientific, Waltham, MA, USA). qRT-PCR was performed using the PowerUP SYBR Green Master Mix (Thermo Fisher Scientific) with mRNA-specific primers (Takara Bio, Shiga, Japan) for *α-SMA*, *COL1A2*, *FN1*, *TGFBR2*, *FOXO3*, *TGIF1*, *CDC42BPB*, *EPO*, *HIF3A*, *SOD2*, *CAT*, *BNIP3*, *ATG12*, *LC3*, *CTSL*, *BCL2*, *FASL*, *TRAIL*, *BBC3*, *RBL2*, *CDKN1B*, *PEPCK*, *G6PC*, and *FBXO32*. The QuantStudio 12K Flex Real-Time PCR system (Thermo Fisher Scientific) was used as the gene amplification device. U6 small nuclear 2 (*RNU6-2*) was used as an endogenous control for miRNA and glyceraldehyde-3-phosphate dehydrogenase (*GAPDH*) was used as an endogenous control for mRNA. Expression levels were calculated by 2^−ΔΔCT^ and compared between the groups.

### 4.5. miR−122−5p Mimic and miR−122−5p Inhibitor

The miR−122−5p mimic was purchased from Sigma−-Aldrich (St Louis, MO, USA). The miR−122−5p inhibitor and control miRNA were purchased from GeneDesign Inc. (Osaka, Japan). The sequences of the miR−122−5p mimic are 5′-[AmC6] CAAACACCAUUGUCACACUUCCATT-3′ (sense) and 5′-UGGAGUGUGACAAUGGUGUUUGTT-3′ (antisense). The sequences of the miR−122−5p inhibitor are 5′-GACGGCGCUAGGAUCAUCAACCAAACACCAUUGUCACACUCCACAAGUAUUCUGGU-3′ (sense) and 5′-ACCAGAAUACAACCAAACACCAUUGUCACACUCCACAAGAUGAUCCUAGCGCCGUC-3′ (antisense).

The sequences of the control miRNA are 5′-[AmC6] UGAACAGUGUACGUACGAUACC[dT][dT]-3′ (sense) and 5′-GGUUCGUACGUACACUGUUCA[dT][dT]-3′ (antisense).

### 4.6. Delivery of the miRNA Mimic and miRNA Inhibitor to Kidneys

PEI-NPs were purchased from Polyplus-transfection SA (Illkirch-Graffenstaden, France). PEI-NPs can be used as a non-viral vector for the delivery of miRNA mimic and miRNA inhibitors to kidney tissues in vivo [9]. PEI-NPs were administered at 150 mmol/L (expressed as the concentration of nitrogen residues) in sterile water provided by the manufacturer. The volume of PEI-NPs was prepared according to the amount of nucleic acid to be delivered to the kidney (N/P ratio, described later) according to the manufacturer’s instructions. The mechanism by which PEI-NP acts as a vector is as follows: (i) PEI-NP forms a cationic complex with oligonucleic acid; (ii) the cationic complex adheres to the cell surface and undergoes endocytosis; and (iii) the oligonucleic acids are translocated into the nucleus by the pH buffering effect in endosomes [68,69,70]. We previously confirmed that PEI-NPs delivered nucleic acids to kidney tissues by assays using Cy3-labeled miRNA in vivo [9]. The N/P ratio reflects the number of PEI-NP nitrogen residues per nucleic acid phosphate and was reported to be associated with the stability and cytotoxicity of the nucleic acid-PEI-NP complex [71,72,73]. In this study, the reagents were mixed at N/P = 6 according to the manufacturer’s instructions. C57BL/6 mice were randomly divided into five treatment groups: Sham group (n = 4), UUO + no injection group (n = 4), UUO + miR−122−5p mimic group (n = 4), UUO + miR−122−5p inhibitor group (n = 4), or UUO + control miRNA group (n = 4). miR−122−5p mimic-PEI-NPs (5 nmol miRNA mimic, N/P ratio 6), miR−122−5p inhibitor–PEI−NPs (5 nmol miRNA inhibitor, N/P ratio 6), and control miRNA–PEI−NPs (5 nmol control miRNA, N/P ratio 6) were dissolved in 200 µL of 5% glucose solution and injected via the tail vein. Injections were performed on the day before surgery (day −1) and days 1, 3, and 6 after UUO surgery or sham surgery. The mice were euthanized on day 10 after surgery. The kidneys were surgically collected and washed with phosphate-buffered saline. 

### 4.7. Kidney Histology (AZAN Staining and Sirius Red Staining)

AZAN staining and Sirius red staining were conducted to evaluate the severity of renal fibrosis. Surgically collected pieces of kidney tissue were fixed in 4% paraformaldehyde and then embedded in paraffin. The tissues were sectioned at 4 µm thick, deparaffinized, and rehydrated. For AZAN staining, kidney sections were exposed to the AZAN staining-specific mordant (Muto Pure Chemicals, Tokyo, Japan) for 10 min and then stained with Mallory’s azocarmin G stain solution (Muto Pure Chemicals) for 90 min. The sections were incubated with 5% phosphotungstic acid solution (Muto Pure Chemicals) for 60 min and stained with Mallory’s aniline blue orange G stain solution (Muto Pure Chemicals) for 60 min. For Sirius red staining, kidney sections were stained with Sirius red mixture [(ratio of 100 mL of Van Gieson’s stain solution A (Muto Pure Chemicals) and 4 mL of 1% Sirius red solution (Muto Pure Chemicals)] for 60 min. The stained kidney sections were washed with distilled water, dehydrated, and mounted for microscopy using BZ-X710 (Keyence, Osaka, Japan). For the quantitative evaluation of renal fibrosis, the area stained red with Sirius red staining was measured. Six images were obtained at 200× magnification in random fields. The red area was measured using Keyence Hybrid Cell Count on a BZ-X Analyzer (version 1.4.0, Keyence). For randomization, digital images of kidney sections were segmented, numbered in sequence, and selected using a random number generator [https://www.random.org/ (accessed on 12 June 2020)].

### 4.8. IHC

IHC was conducted to evaluate the protein levels of TGFBR2 and FOXO3. Surgically collected pieces of kidney tissue were fixed in 4% paraformaldehyde and then embedded in paraffin. The tissues were sectioned at 4 µm thick, deparaffinized, and rehydrated. After antigen activation and blocking, kidney sections were incubated overnight at 4 °C with a TGFBR2-specific primary antibody (Fitzgerald Industries International, Acton, MA, USA) and FOXO3-specific primary antibody (Bethyl, Waltham, MA, USA). After washing, they were incubated with peroxidase-conjugated secondary antibodies for 1 h at room temperature (NICHIREI, Tokyo, Japan). Then, kidney sections were incubated with ImmPACT DAB Substrate Kit (Vector, Newark, CA, USA) for 10 min at room temperature, incubated in Meyer hematoxylin solution (Muto Pure Chemicals) for 10 s, washed, and dehydrated. Kidney sections were observed using a BZ-X710 (Keyence). The IHC positive area (brown stain) was quantified. Six images from random fields were obtained at 200× magnification. The brown stained area was measured using Keyence Hybrid Cell Count on a BZ-X Analyzer (version 1.4.1.1, Keyence). For randomization, digital images of kidney sections were segmented, numbered in sequence, and selected using a random number generator [https://www.random.org/ (accessed on 15 October 2022)].

### 4.9. Measurement of Serum BUN

The measurement of serum BUN was conducted by SRL (Tokyo, Japan).

### 4.10. Nomenclature System for miRNAs

The notation of miRNAs follows the guidelines for the nomenclature system for miRNAs [74].

### 4.11. Statistical Analysis

All values are expressed as the means ± standard error obtained from two independent experiments. Statistical comparisons between two groups were made using the Student’s *t* test. Comparisons between groups of three or more were made by one-way analysis of variance (ANOVA) and Newman–Keuls method. The analyses shown in Figure 4, Figure 5, Figure 6, Figure 7, Figure 8 and Figure 9 compared the four treatment groups (excluding sham group). This statistical method was used because the large difference in the results between the sham group and other groups might affect the analysis of the effects of miR−122−5p on renal fibrosis. *p* < 0.05 was considered statistically significant. The analysis of the comparison between the sham group and UUO + injection group is shown in Supplementary Figures S1–S7.

## Figures and Tables

**Table 1:** miRNAs involved renal fibrosis.

microRNA	Sequences	Fold Change	*p*
		(UUO Mice/Control Mice)	
miR−511−3p	AAUGUGUAGCAAAAGACAGGAU	312.3	<0.01
miR−375−3p	UUUGUUCGUUCGGCUCGCGUGA	143.0	<0.01
miR−130b−3p	CAGUGCAAUGAUGAAAGGGCAU	61.8	<0.01
miR−127−3p	UCGGAUCCGUCUGAGCUUGGCU	55.7	<0.01
miR−466b−3p	AUACAUACACGCACACAUAAGA	48.8	<0.01
miR−7211−3p	UGGAGUGACUGUAGGGAGGAUGC	41.7	<0.01
miR−669l−5p	AGUUGUGUGUGCAUGUAUAUGU	40.6	<0.01
miR−214−5p	UGCCUGUCUACACUUGCUGUGC	39.1	<0.01
miR−6929−3p	AGGGAGGAGCAGCAUCUGUGA	38.9	<0.01
miR−467b−5p	GUAAGUGCCUGCAUGUAUAUG	35.5	<0.01
miR−8109	CCCGCGGCCG	33.4	<0.01
miR−467a−3p	TGTAGGTGTGTGTATGTATA	33.3	<0.01
miR−136−5p	CCATCATCAAAACAAATGGAGT	30.9	<0.01
miR−421−3p	GCGCCCAATTAATGTCTG	26.0	<0.01
miR−467e−5p	ACATATACATGCTCACACT	25.6	<0.01
miR−290b−3p	TACTCAAACTATGGGGGC	19.0	<0.01
miR−1907	ACCTCCAGATCCTCTG	17.7	<0.01
miR−1967	GCATCTTCTCCCCAG	17.7	<0.01
miR−7687−5p	AGCCTGCGCCTCA	17.4	<0.01
miR−106b−3p	GCAGCAAGTACCCAC	15.1	<0.01
miR−379−5p	CCTACGTTCCATAGTC	14.5	<0.01
miR−3547−5p	TCCCGGGCCCC	14.1	<0.01
miR−1947−3p	GGAGGGAGAGCTAGC	13.6	<0.01
miR−3091−5p	GCGGGCCCAACC	13.5	<0.01
miR−134−5p	CCCCTCTGGTCAA	13.2	<0.01
miR−31−3p	GATGGCAATATGTTGGCAT	13.2	<0.01
miR−5099	GGAGCACCACATCG	11.4	<0.01
miR−141−5p	TCCAACACTGCACTGGA	10.9	<0.01
miR−15b−3p	TAGAGCAGCAAATAATGATTCG	10.9	<0.01
miR−300−3p	GAAGAGAGCTTGCCCTTG	10.8	<0.01
miR−342−5p	CTCAATCACAGATAGCACC	10.4	<0.01
miR−8095	GGGACAGACAGCAGA	10.2	<0.01
miR−6984−3p	AGAAAGACAGGAAAGAAAG	10.0	<0.01
miR−714	GCGACCGACCGGCC	9.6	<0.01
miR−3472	GGTTCCTTCCAGCTT	9.5	<0.01
miR−1957a	GTCATATGCTCTACCACT	9.4	<0.01
miR−5131	GCTCGGGGCTCC	8.8	<0.01
miR−7047−3p	GGAAGGAGGAAGGGT	8.6	<0.01
miR−3473c	CTTATTATGGGGGCTGG	8.1	<0.01
miR−21a−3p	GACAGCCCATCGACT	7.8	<0.01
miR−6392−3p	AGAGGACCCGGCA	7.2	<0.01
miR−710	CTCAACTCTCCCCA	7.0	<0.01
miR−7046−5p	CCTGGCTCCCAGC	6.9	<0.01
miR−146b−5p	AGCCTATGGAATTCAGTTC	6.4	<0.01
miR−770−3p	CCAGCTCCACGTC	6.0	<0.01
miR−6961−5p	GGCCATTTCCTTCCC	5.6	<0.01
miR−376a−3p	ACGTGGATTTTCCTCTA	5.5	<0.01
miR−6354	AGAGACCTGATCCCCA	5.3	<0.01
miR−1931	GCCATCGCACCAGC	5.2	<0.01
miR−501−3p	CAAATCCTTGCCCGG	4.9	<0.01
miR−21a−5p	TCAACATCAGTCTGATAAGC	4.9	<0.01
miR−132−3p	CGACCATGGCTGTAGA	4.9	<0.01
miR−468−3p	CAGACACACGCACATCA	4.7	<0.01
miR−214−3p	ACTGCCTGTCTGT	4.4	<0.01
miR−7672−5p	TCGCCCGCTGTCA	4.4	<0.01
miR−292b−3p	ATACTCAAACTGGGGGC	4.0	<0.01
miR−7063−5p	TGTGCTCAGCCTGC	4.0	<0.01
miR−5132−5p	CCTGAGTCCACCACC	4.0	<0.01
miR−3093−3p	CCAACCTCCCACGG	4.0	<0.01
miR−142a−3p	TCCATAAAGTAGGAAACACTACA	4.0	<0.01
miR−5121	GGAGATGTCTCATCACA	4.0	<0.01
miR−223−3p	TGGGGTATTTGACAAACTGAC	3.9	<0.01
miR−224−5p	AACGGAACCACTAGTGACTTA	3.9	<0.01
miR−3572−5p	GTCCACCTTGCCCT	3.8	<0.01
miR−6963−5p	CCAGGTTCTGCCATC	3.8	<0.01
miR−6921−5p	GCTTCCTACCTCATGC	3.7	<0.01
miR−467a−5p	CGCATATACATGCAGGCA	3.5	<0.01
miR−6349	CGCATGCCCCTCC	3.5	<0.01
miR−669b−5p	ACATGCACATGCACACA	3.5	<0.01
miR−299b−5p	ATGTATGTGGGACGGTAAAC	3.5	<0.01
miR−7080−3p	AGGGAACGGAGGGG	3.5	<0.01
miR−142a−5p	AGTAGTGCTTTCTACTTTA	3.4	<0.01
miR−199a−5p	GAACAGGTAGTCTGAACAC	3.2	<0.01
miR−8093	CACTCATGCTCTGCTC	3.1	<0.01
miR−183−5p	AGTGAATTCTACCAGTGCC	3.1	<0.01
miR−7040−5p	CGCCTCCATCTCCC	3.0	<0.01
miR−718	CGACACCCGGCCG	3.0	<0.01
miR−7235−5p	GCCCAGACCCCTC	3.0	<0.01
miR−762	GCTCTGTCCCGGC	3.0	<0.01
miR−199a−3p	TAACCAATGTGCAGACTACT	3.0	<0.01
miR−672−5p	TCACACACAGTACACCA	2.9	<0.01
miR−18a−5p	CTATCTGCACTAGATGCAC	2.8	<0.01
miR−3067−3p	CCTCTCCCAGGGC	2.8	<0.01
miR−292a−5p	CAAAAGAGCCCCCAG	2.7	<0.01
miR−342−3p	ACGGGTGCGATTTCTGT	2.7	<0.01
miR−20a−3p	CTTTAAGTGCTCGTAATGCA	2.7	<0.01
miR−let−7i−3p	AGCAAGGCAGTAGCTT	2.6	<0.01
miR−290a−3p	GGGCTTAAAACTAGGCGGC	2.6	<0.01
miR−182−5p	CGGTGTGAGTTCTACC	2.5	<0.01
miR−149−3p	GCACCGCCCCC	2.5	<0.01
miR−877−5p	CCCTGCGCCATCT	2.5	<0.01
miR−1949	AACTATGCTGACATCCTG	2.5	<0.01
miR−674−5p	TACACCACTCCCAT	2.5	<0.01
miR−199b−5p	GAACAGGTAGTCTAAACACTGG	2.5	<0.01
miR−31−5p	CAGCTATGCCAGCATCT	2.4	<0.01
miR−125a−3p	GGCTCCCAAGAACCTC	2.4	<0.01
miR−721	TTCCCCCTTTTAATT	2.4	<0.01
miR−7115−3p	CTGTGGGGGCAGG	2.3	<0.01
miR−3474	GAATCCACGTCTCCTC	2.3	<0.01
miR−28a−3p	TCCAGCAGCTCACA	2.2	<0.01
miR−3075−5p	GTCCTTGGCTGCTC	2.2	<0.01
miR−5126	CCCCGCCCCCG	2.2	<0.01
miR−155−5p	ACCCCTATCACAATTAGC	2.1	<0.01
miR−2861	CCGCCCGCCG	2.1	<0.01
miR−5128	AGCCATCTCGCCAGC	2.1	<0.01
miR−135a−1−3p	CGCCACGGCTCCA	2.1	<0.01
miR−8110	CCCCCCCCCCA	2.1	<0.01
miR−211−3p	GCCCCCCTTTGCT	2.0	<0.01
miR−7118−5p	GTTCCCTCTCCCGC	2.0	<0.01
miR−6975−5p	GCUGGGGAGAAAGGGGUUUGGCA	−253.2	<0.01
miR−6918−5p	UGCUGAGGACGGGAUUAGGUUCU	−252.2	<0.01
miR−6904−5p	UCCUGGGGUUAGAGUUGAGUGG	−194.5	<0.01
miR−122−5p	UGGAGUGUGACAAUGGUGUUUG	−183.2	<0.01
miR−7682−3p	CCUGUGGGUUGGGUUGGCUUU	−164.8	<0.01
miR−129b−5p	GCUUUUUGGGGUAAGGGCUUCC	−134.9	<0.01
miR−190b−5p	UGAUAUGUUUGAUAUUGGGUUG	−107.9	<0.01
miR−504−3p	AGGGAGAGCAGGGCAGGGUUUC	−104.0	<0.01
miR−363−3p	AAUUGCACGGUAUCCAUCUGUA	−89.6	<0.01
miR−7218−5p	UGCAGGGUUUAGUGUAGAGGG	−85.3	<0.01
miR−8119	GACCCTAGCTCCCTC	−70.6	<0.01
miR−7080−5p	CCAAACCCACCTCC	−67.1	<0.01
miR−1927	TCAGTCCCTAACATCCA	−57.8	<0.01
miR−6990−5p	AGAGCCCTGACTCACC	−56.3	<0.01
miR−7234−3p	CCTTCTACCCTAGAAAGA	−51.7	<0.01
miR−6905−5p	TCATTCAACCCAACCTG	−49.6	<0.01
miR−7055−5p	CCAACTCAGATAACCCA	−46.6	<0.01
miR−190a−3p	AGGAATATGCTTGATATATAGT	−42.8	<0.01
miR−6401	ACCCGACACCACTG	−42.2	<0.01
miR−7233−5p	CATCTATCTGTCCCTAACT	−41.4	<0.01
miR−7657−5p	TTACCTAACTATCCAACTATT	−36.4	<0.01
miR−3078−3p	CCTAAAGACTACCCCAG	−35.4	<0.01
miR−7664−3p	ATTAGTTAACCCAGCCTAA	−33.4	<0.01
miR−7086−5p	TGCCCAAACCTTTCTC	−33.1	<0.01
miR−6902−3p	CTGAACCCACACATCA	−32.7	<0.01
miR−188−3p	TGCAAACCCTGCATGTG	−31.2	<0.01
miR−7028−5p	CTCCTGACCCAAGC	−27.7	<0.01
miR−6481	CATCTAAGCATTTTCAGTG	−25.8	<0.01
miR−302c−5p	GCAGGTAACCCCAT	−24.6	<0.01
miR−6980−5p	CTAACCTAGCCTCCCC	−24.2	<0.01
miR−467b−3p	GTGTTGGTGTGTGTAT	−23.8	<0.01
miR−7219−3p	AGTGTGTTAGAAACCCG	−21.1	<0.01
miR−194−2−3p	CAGATAACAGCAGCCC	−19.6	<0.01
miR−7074−5p	ACTGGAGCCCTAGCC	−19.0	<0.01
miR−6998−5p	AGTCACTTTGCCCTCT	−18.2	<0.01
miR−874−5p	CTTACCCTGGTGCG	−17.4	<0.01
miR−6923−5p	ACACCCCAATCCTCC	−16.4	<0.01
miR−327	ATCCTCATGCCCCT	−15.7	<0.01
miR−1188−3p	GCAGGGTGTGGTGG	−15.7	<0.01
miR−3070−2−3p	TCTACCCCTGACCATAG	−14.9	<0.01
miR−101a−5p	GCATCAGCACTGTGAT	−10.9	<0.01
miR−3070−3p	TCTACCCCTGACGGT	−10.5	<0.01
miR−6926−5p	TCACCATCCCTCACC	−8.8	<0.01
miR−6981−5p	GCCTTCAGCCTCTTC	−8.6	<0.01
miR−3063−3p	GGCGAGAGATCAGGA	−8.6	<0.01
miR−6236	CCTGACTGCCGGC	−7.2	<0.01
miR−30c−1−3p	GGAGTAAACAACCCTCTCC	−6.0	<0.01
miR−883b−5p	TGACTGCTACCCATT	−5.6	<0.01
miR−26b−3p	GAGCCAAGTAATGGAGAACA	−5.3	<0.01
miR−744−3p	AGGTTGAGGTTAGTGGCA	−5.1	<0.01
miR−6988−5p	TGGGCCTCAGCTCT	−4.9	<0.01
miR−376b−3p	AAGTGGATGTTCCTCTAT	−4.3	<0.01
miR−6393	ACTCAGTGTGCTTCGT	−4.1	<0.01
miR−7094b−2−5p	TCAGACCCTGTATCCTC	−3.9	<0.01
miR−7056−5p	AACCTCTCTGTCCTCC	−3.9	<0.01
miR−6971−5p	AGCCTCTACACCCTCC	−3.9	<0.01
miR−497b	CCACGTCCAAACCA	−3.9	<0.01
miR−874−3p	TCGGTCCCTCGGG	−3.6	<0.01
miR−192−3p	CTGTGACCTATGGAATTG	−3.5	<0.01
miR−873b	GTGTGCATTTGCAGGA	−3.5	<0.01
miR−7075−5p	AAAACCATGTCCTCCTC	−3.4	<0.01
miR−703	TTCTTTCCTTCTGAAGGTT	−3.3	<0.01
miR−193a−5p	TCATCTTGCCCGCA	−3.3	<0.01
miR−7670−5p	TTCCCAATCTGCCCA	−3.2	<0.01
miR−8094	TCTTCTCGTTGTCCTTC	−3.2	<0.01
miR−144−5p	ACTTACAGTATATGATGATATCC	−3.2	<0.01
miR−669c−3p	TTTACTTGTGTGTGTGTG	−3.0	<0.01
miR−193a−3p	ACTGGGACTTTGTAGGC	−3.0	<0.01
miR−190a−5p	ACCTAATATATCAAACATATCA	−3.0	<0.01
miR−30e−5p	CTTCCAGTCAAGGATGT	−2.9	<0.01
miR−302c−3p	CCACTGAAACATGGAAGCAC	−2.8	<0.01
miR−192−5p	GGCTGTCAATTCATAGGTC	−2.7	<0.01
miR−30e−3p	GCTGTAAACATCCGACTG	−2.7	<0.01
miR−681	AGCTGCCTGCCAG	−2.6	<0.01
miR−376c−3p	ACGTGAAATTTCCTCTATGTT	−2.6	<0.01
miR−466g	TGTGTGTGCATGTGTC	−2.6	<0.01
miR−30c−5p	GCTGAGAGTGTAGGATGT	−2.6	<0.01
miR−194−5p	TCCACATGGAGTTGCT	−2.6	<0.01
miR−802−5p	AAGGATGAATCTTTGTTACTGA	−2.6	<0.01
miR−365−3p	ATAAGGATTTTTAGGGGCATTA	−2.5	<0.01
miR−378a−5p	ACACAGGACCTGGAGTCA	−2.5	<0.01
miR−7211−5p	GGTGGAGTGGCAGA	−2.5	<0.01
miR−5113	ACAGGATCTCTCTCCTC	−2.5	<0.01
miR−378b	TCTTCTGACTCCAAGTC	−2.5	<0.01
miR−29c−5p	GAACACCAGGAGAAATCGGTC	−2.4	<0.01
miR−378d	ACCTTCTGACTCCAAGG	−2.4	<0.01
miR−185−5p	TCAGGAACTGCCTTTCT	−2.4	<0.01
miR−378a−3p	CCTTCTGACTCCAA	−2.4	<0.01
miR−7219−5p	TCTCAACCCTGAGCTC	−2.3	<0.01
miR−29c−3p	TAACCGATTTCAAATGGTGCTA	−2.3	<0.01
miR−378c	GCTTCTGACTCCAAGT	−2.3	<0.01
miR−3473b	CTGAGCCATCTCTCCA	−2.3	<0.01
miR−30a−3p	GCTGCAAACATCCGACT	−2.3	<0.01
miR−let−7f−1−3p	GGGAAGGCAATAGATTGTAT	−2.3	<0.01
miR−203−3p	CTAGTGGTCCTAAACATT	−2.3	<0.01
miR−101c	TCAGTTATCACAGTACTGT	−2.2	<0.01
miR−129−1−3p	ATACTTTTTGGGGTAAGGG	−2.2	<0.01
miR−144−3p	AGTACATCATCTATACTGTA	−2.2	<0.01
miR−455−5p	CGATGTAGTCCAAAGGCA	−2.2	<0.01
miR−451a	AACTCAGTAATGGTAACGGTTT	−2.2	<0.01
miR−3095−3p	AAAAGCTCTCTCTCCAGT	−2.2	<0.01
miR−690	TTTGGTTGTGAGCCTA	−2.2	<0.01
miR−455−3p	GTGTATATGCCCGTGG	−2.1	<0.01
miR−466q	ACGTATGTGTGTGTGTG	−2.1	<0.01
miR−3099−3p	TCCCCAACCTCTCTC	−2.1	<0.01
miR−760−3p	TCCCCACAGACCCA	−2.1	<0.01
miR−3473a	TGCTGAGCCATCTCTC	−2.1	<0.01
miR−30a−5p	CTTCCAGTCGAGGATGT	−2.1	<0.01
miR−29b−1−5p	TAAACCACCATATGAAACCAGC	−2.1	<0.01
miR−664−5p	CCAGTCATTTTCCCCA	−2.0	<0.01
miR−30c−2−3p	AGAGTAAACAGCCTTCTCC	−2.0	<0.01
miR−33−5p	TGCAATGCAACTACAATGCAC	−2.0	<0.01
miR−295−5p	GAAGTGTGCCCCAC	−2.0	<0.01

Abbreviations: miRNA, microRNA; UUO, unilateral ureteral obstruction.

**Table 2:** SOD2 and LC3 in human CKD and its causative diseases.

Target	Authors	Country	Year	Patients and Number	Results and Findings	References
SOD2	Möllsten et al.	Sweden	2009	Type I DM patients (n = 411)	Genetic *SOD2* polymorphisms were associated with the development of DM nephropathy caused by type Ⅰ DM.	[51]
SOD2	Prunotto et al.	Italy	2010	MN patient (n = 24)	Anti-SOD2 antibodies were specifically detected in the serum of primary MN patients. Anti-SOD2 antibodies were deposited in the glomerular podocytes of MN patients.	[52]
SOD2	Olsson et al.	Sweden	2011	CKD patients (n = 30)	Neutrophils from CKD patients were stimulated with lipopolysaccharide. *SOD2* gene expression was decreased in the neutrophils of CKD patients but not controls.	[53]
SOD2	Zaza et al.	Italy	2013	PD patients (n = 15)	Plasma malondialdehyde (an oxidative marker) levels were higher in PD patients compared with controls, leading to increased *SOD2* gene expression.	[54]
SOD2	Mohammedi et al	France	2014	Type Ⅰ DM patients (n = 1285)	*SOD2* gene mutations were associated with the onset and progression of DM nephropathy, plasma advanced oxidation protein product (an oxidative marker) concentrations, and antioxidant activity in type Ⅰ DM patients.	[55]
SOD2	Krueger et al.	Denmark Germany	2016	CKD patients (n = 120) HD patients (n = 81)	SOD2 protein levels in monocytes decreased as the CKD stage progressed. After HD induction, SOD2 protein levels began to increase.	[56]
SOD2	Jerotic et al.	Serbia.	2019	HD patients (n = 256)	Genetic *SOD2* polymorphisms were associated with risk of end-stage renal disease.	[57]
SOD2	Corredor et al.	Spain	2020	CKD patients (n = 548)	Genetic *SOD2* polymorphisms were associated with the erythropoietin resistance of renal anemia.	[58]
LC3	Miyazaki et al.	Japan	2014	IgAN patients (n = 48)	In IgAN patients, prorenin receptor expression was an autophagy (LC3)-mediated compensatory response to IgAN progression.	[59]
LC3	Xiong et al.	USA	2019	DM nephropathy patients (n = 12)	LC3 protein obtained from urinary stem cells in DM nephropathy patients was decreased compared with the control group.	[60]
LC3	Liu et al.	China	2019	DM nephropathy patients (n = 11)	Podocytes of DM nephropathy patients had more LC3 positive puncta compared with the control group.	[61]
LC3	Ogawa-Akiyama et al.	Japan	2020	MCNS patients (n = 41) MN patients (n = 37)	LC3 was localized to glomerular podocytes, suggesting autophagy mainly occurred in the glomerular podocytes of MCNS patients.	[62]
LC3	da Silva et al.	Brazil	2020	FSGS patients (n = 22) MCNS patients (n = 27)	LC3-positive glomerular podocytes were denser in MCNS patients than in FSGS patients.	[63]

Abbreviations: SOD2, superoxide dismutase 2, mitochondrial; LC3, microtubule-associated proteins 1A/1B light chain 3B, DM, diabetes mellitus; MN, membranous nephropathy; CKD, chronic kidney disease; PD, peritoneal dialysis; HD, hemodialysis; IgAN, immunoglobulin a nephropathy; MCNS, minimal change nephrotic syndrome; FSGS, focal segmental glomerulosclerosis.

## Data Availability

Data are available on request.

## Supplementary Materials

The following supporting information can be downloaded at: https://www.mdpi.com/article/10.3390/ijms232315423/s1. Figure S1. Modulation of miR−122−5p expression after UUO surgery (10 days after surgery). The expression levels of miR−122−5p were determined by qRT-PCR. Relative expression levels are shown as mean values and standard errors. Relative expression levels were compared between the sham group (n = 4) and UUO + no injection group (n = 4). Abbreviations: miR, microRNA; UUO, unilateral ureteral obstruction; qRT-PCR, quantitative real-time reverse-transcription polymerase chain reaction; NS, not significant. Figure S2. The effects of UUO surgery on mRNAs (*COL1A2*, *FN1*, and *α-SMA*). The expression levels of *COL1A2*, *FN1*, and *α-SMA* were determined by qRT-PCR. Relative expression levels are shown as mean values and standard errors. Relative expression levels of mRNAs were compared between the sham group (n = 4) and UUO + no injection group (n = 4). Abbreviations: miR, microRNA; UUO, unilateral ureteral obstruction; mRNA, messenger RNA; COL1A2, collagen 1A2; FN1, fibronectin 1; α-SMA, α-smooth muscle actin; qRT-PCR, quantitative real-time reverse-transcription polymerase chain reaction. * *p* < 0.05. Figure S3. Kidney histology. Renal fibrosis areas were evaluated by Sirius red staining. Fibrotic areas were compared between the sham group (n = 6) and UUO + no injection group (n = 6). Abbreviations: miR, microRNA; UUO, unilateral ureteral obstruction. * *p* < 0.05. Figure S4. Serum blood urea nitrogen concentrations. Serum blood urea nitrogen concentrations (mg/dL) were compared between the sham group (n = 4) and UUO + no injection group (n = 4). Abbreviations: miR, microRNA; UUO, unilateral ureteral obstruction. * *p* < 0.05. Figure S5. The effects of UUO surgery on fibrosis-related mRNAs. The expression levels of six mRNAs (*TGFBR2*, *FOXO3*, *TGIF1*, *CDC42BPB*, *EPO*, and *HIF3A*) were determined by qRT-PCR. Relative expression levels are shown as mean values and standard errors. Relative expression levels of mRNAs were compared between the sham group (n = 4) and UUO + no injection group (n = 4). Abbreviations: miR, microRNA; mRNA, messenger RNA; UUO, unilateral ureteral obstruction; qRT-PCR, quantitative real-time reverse-transcription polymerase chain reaction; TGFBR2, transforming growth factor, beta receptor II; FOXO3, forkhead box O3; TGIF1, Homeobox protein TGIF1; CDC42BPB, CDC42 binding protein kinase beta; EPO, erythropoietin; HIF3A, hypoxia-inducible factor 3 alpha; NS, not significant. * *p* < 0.05. Figure S6. IHC analysis. The protein levels of TGFBR2 and FOXO3 were evaluated by IHC. Comparisons of TGFBR2 and FOXO3 positive areas (brown stain) between the sham group (n = 6) and UUO + no injection group (n = 6) are shown. Abbreviations: IHC, immunohistochemistry; miR, microRNA; UUO, unilateral ureteral obstruction; TGFBR2, trans-forming growth factor, beta receptor II; FOXO3, forkhead box O3. * *p* < 0.05. Figure S7. The effects of UUO surgery on the expression levels of 15 genes downstream of *FOXO3 (SOD2, CAT, BNIP3, ATG12, LC3, CTSL, BCL2, FASL, TRAIL, BBC3, RBL2, CDKN1B, PEPCK, G6PC,* and *FBXO32*). Relative expression levels of genes were compared between the sham group (n = 4) and UUO + no injection group (n = 4). Relative expression levels are shown as mean values and standard errors. * *p* < 0.05.

## Abbreviations

miR, microRNA; UUO, unilateral ureteral obstruction; qRT-PCR, quantitative real-time reverse-transcription polymerase chain reaction; SOD2, superoxide dismutase 2, mitochondrial; CAT, catalase; BNIP, BCL2/adenovirus E1B 19 kDa protein-interacting protein 3; ATG12, autophagy related 12; LC3, microtubule-associated proteins 1A/1B light chain 3B; CTSL, cathepsin L1; BCL2, B-cell lymphoma 2; FASL, fas ligand; TRAIL, TNF-related apoptosis-inducing ligand; BBC3, Bcl-2-binding component 3; RBL2, retinoblastoma-like protein 2; CDKN1B, cyclin-dependent kinase inhibitor 1B; PEPCK, phosphoenolpyruvate carboxykinase; G6PC, glucose-6-phosphatase, catalytic subunit; FBXO32, F-box only protein 32; NS, not significant.
