# Supplementary figures and images for: miR−122−5p Regulates Renal Fibrosis In Vivo
Source: Int J Mol Sci. 2022 Dec 6;23(23):15423. doi: 10.3390/ijms232315423 (PMC9736395; doi:10.3390/ijms232315423)

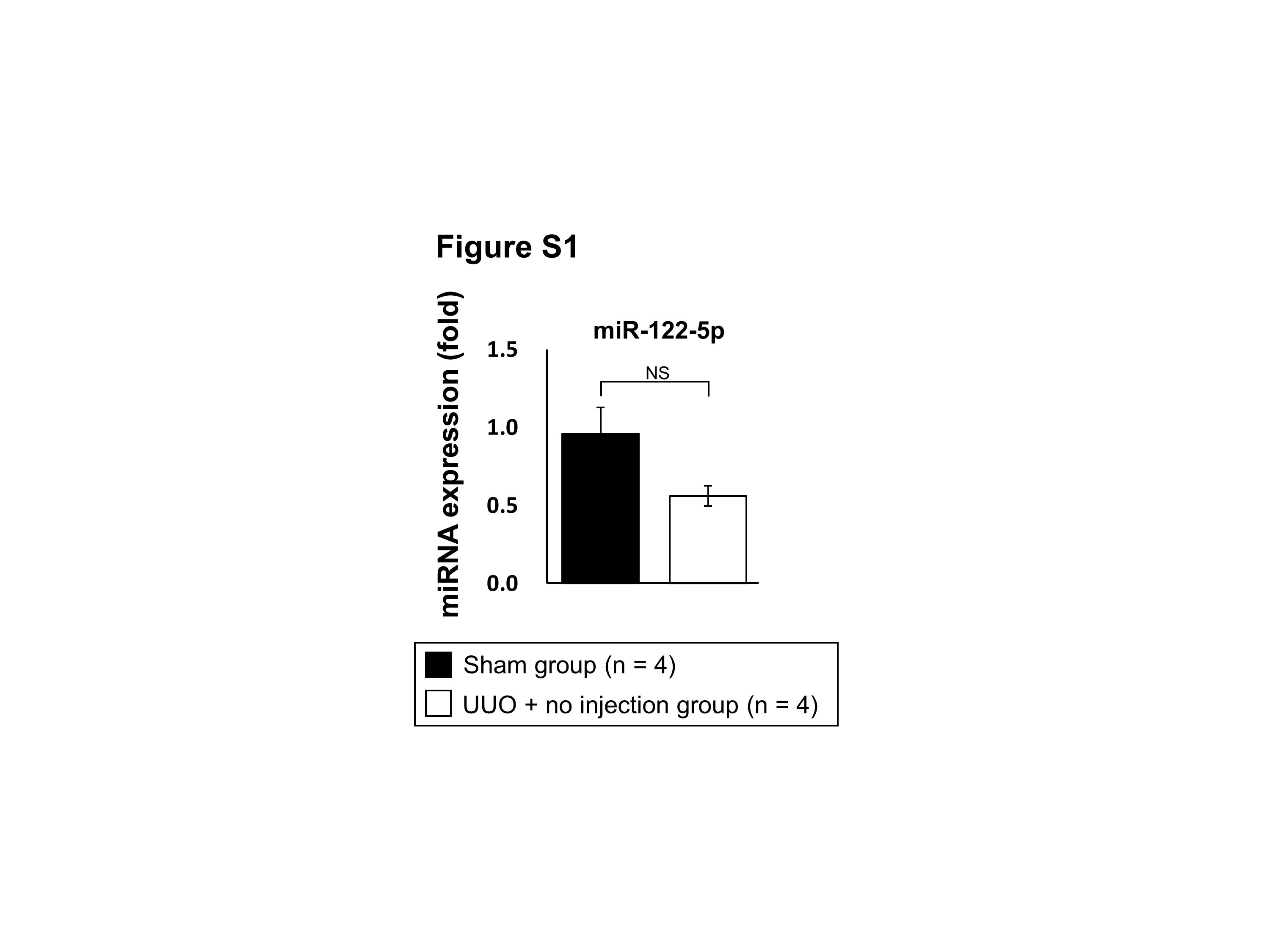

Supplement: Supplementary file 1 [file ijms-23-15423-s001.zip › Supplementary Figure/Figure S1.TIF]

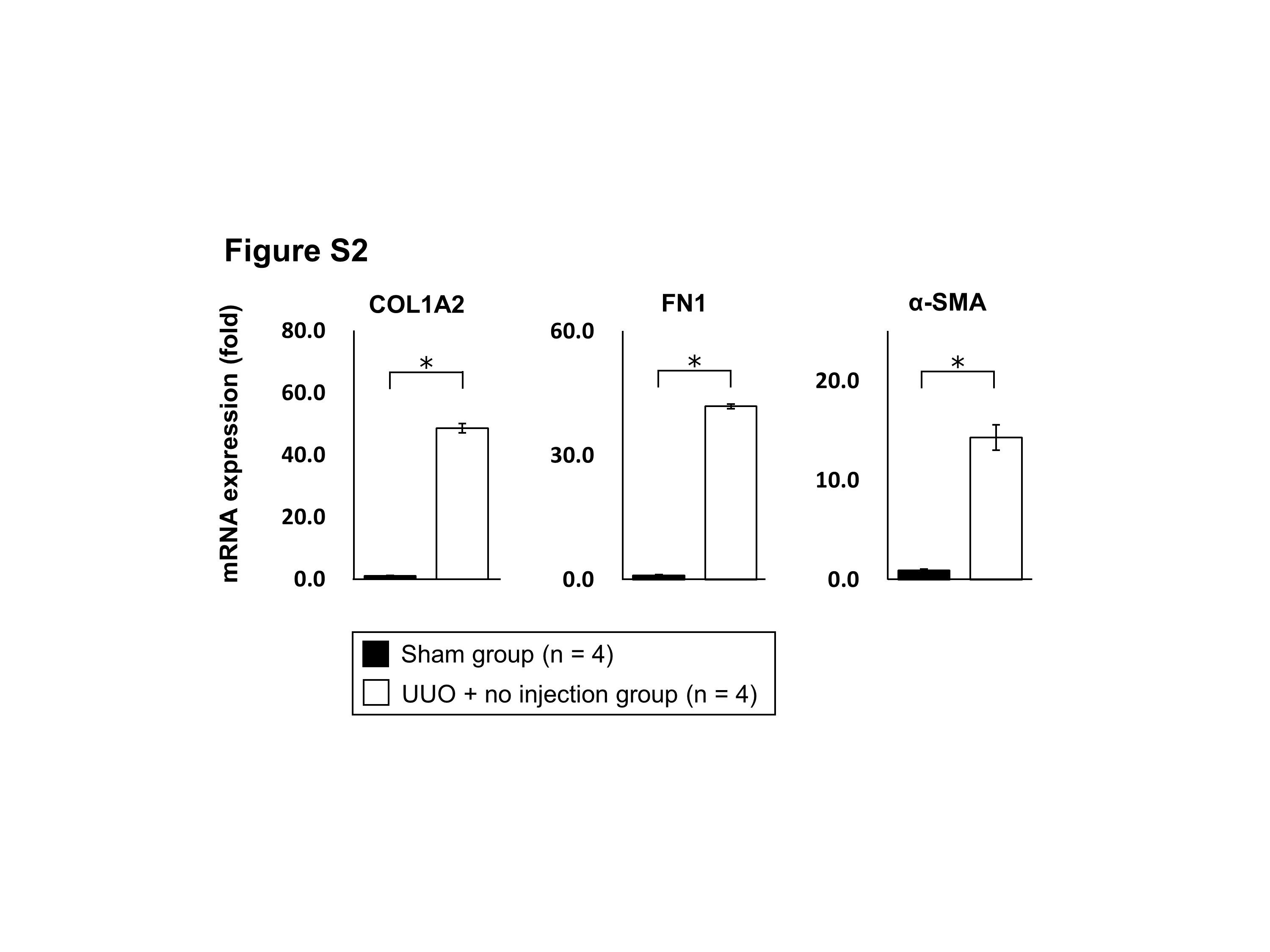

Supplement: Supplementary file 1 [file ijms-23-15423-s001.zip › Supplementary Figure/Figure S2.TIF]

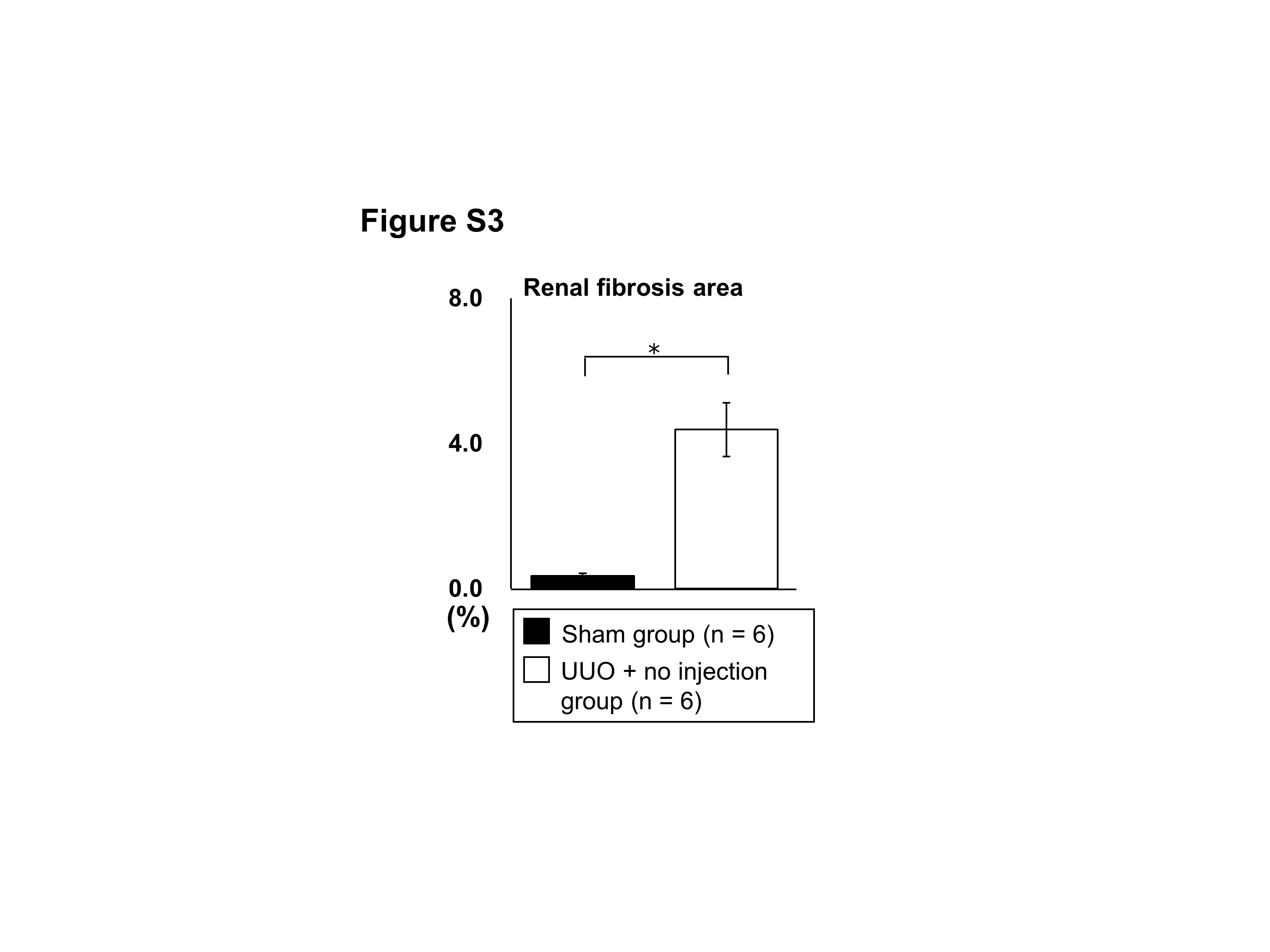

Supplement: Supplementary file 1 [file ijms-23-15423-s001.zip › Supplementary Figure/Figure S3.TIF]

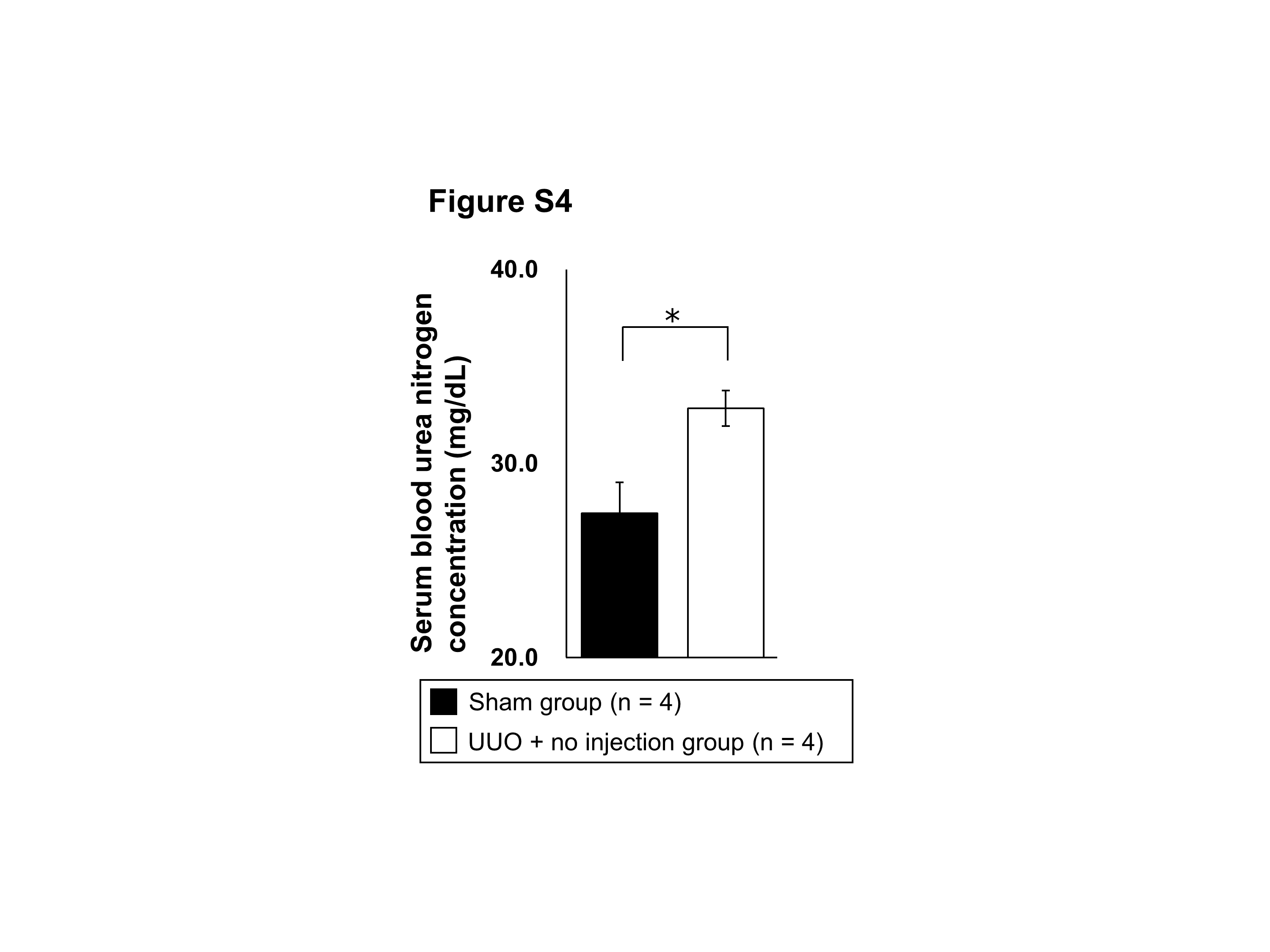

Supplement: Supplementary file 1 [file ijms-23-15423-s001.zip › Supplementary Figure/Figure S4.TIF]

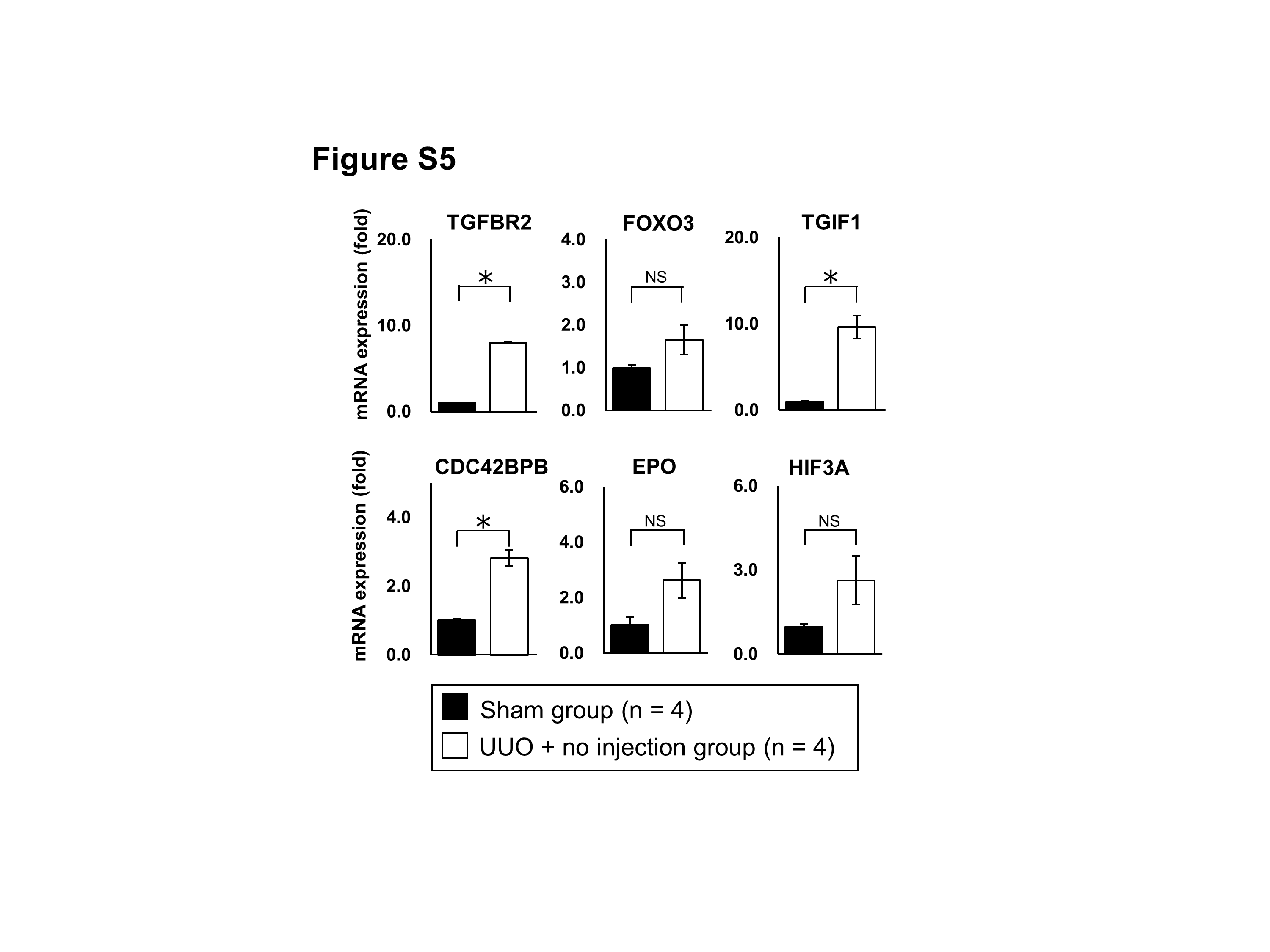

Supplement: Supplementary file 1 [file ijms-23-15423-s001.zip › Supplementary Figure/Figure S5.TIF]

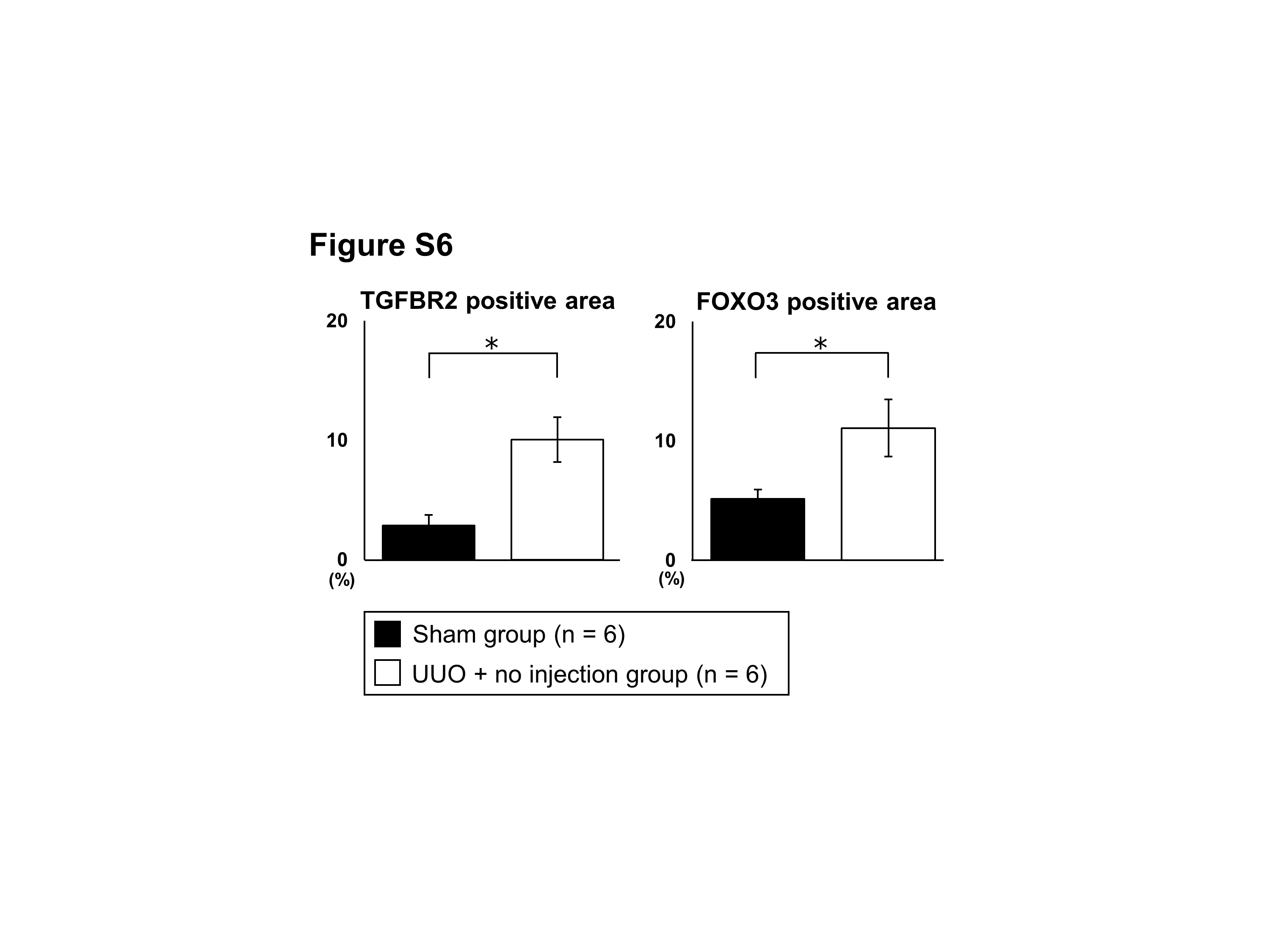

Supplement: Supplementary file 1 [file ijms-23-15423-s001.zip › Supplementary Figure/Figure S6.TIF]

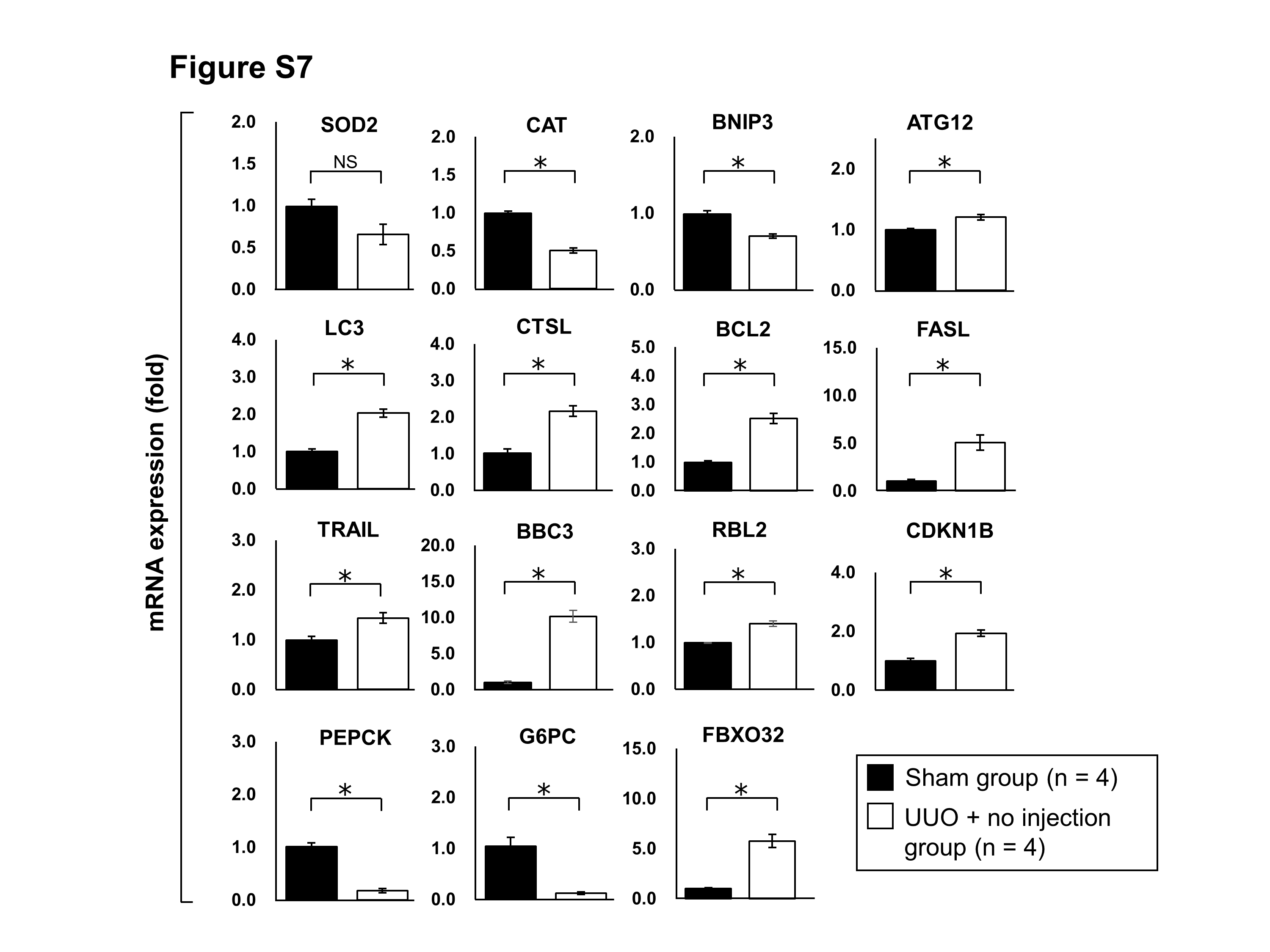

Supplement: Supplementary file 1 [file ijms-23-15423-s001.zip › Supplementary Figure/Figure S7.TIF]
